# Cardiovascular Risk Factors and Differential Transcriptomic Profile of the Subcutaneous and Visceral Adipose Tissue and Their Resident Stem Cells

**DOI:** 10.3390/cells9102235

**Published:** 2020-10-03

**Authors:** Gemma Arderiu, Carmen Lambert, Carlos Ballesta, Fabrizio Moscatiello, Gemma Vilahur, Lina Badimon

**Affiliations:** 1 Cardiovascular-Program ICCC, IR-Hospital Santa Creu I Sant Pau, IIB Sant Pau, 08025 Barcelona, Spain; lambert.goitia@gmail.com (C.L.); gvilahur@santpau.com (G.V.); 2 Ciber CV, 28029 Madrid, Spain; 3 Centro Médico Teknon, 08025 Barcelona, Spain; ballesta@dr.teknon.es (C.B.); fabriziomoscatiello@gmail.com (F.M.); 4 Cardiovascular Research Chair UAB, 08025 Barcelona, Spain

**Keywords:** adipose stem cells, endothelial cells, microvesicles, obesity, cardiovascular risk factors, and angiogenesis

## Abstract

Background: The increase in the incidence of obesity and obesity-related cardiovascular risk factors (CVRFs) over the last decades has brought attention on adipose tissue (AT) pathobiology. The expansion of AT is associated with the development of new vasculature needed to perfuse the tissue; however, not all fat depots have the same ability to induce angiogenesis that requires recruitment of their own endothelial cells. In this study we have investigated the effect of different CVRFs, on the angiogenic capacity of the subcutaneous (SAT) and visceral (VAT) adipose tissue and on the function of their mesenchymal cell reservoir. Methods: A transcriptomic approach was used to compare the different angiogenic and inflammatory profiles of the subcutaneous and visceral fat depots from individuals with obesity, as well as their resident stem cells (ASCs). Influence of other risk factors on fat composition was also measured. Finally, the microvesicles (MVs) released by ASCs were isolated and their regenerative potential analyzed by molecular and cellular methodologies. Results: Obesity decreases the angiogenic capacity of AT. There are differences between SAT and VAT; from the 21 angiogenic-related genes analyzed, only three were decreased in SAT compared with those decreased in VAT. ASCs isolated from both fat depots showed significant differences; there was a significant up-regulation of the VEGF-pathway on visceral derived ASCs. ASCs release MVs that stimulate endothelial cell migration and angiogenic capacity. Conclusions: In patients with obesity, SAT expresses a greater number of angiogenic molecules than VAT, independent of the presence of other CVRFs.

## 1. Introduction

Adipose tissue (AT) is widely distributed all around the organism, and constitute**s** between 15% and 25% of the total body mass; however, due to its plasticity, it can reach up to 40% of the body mass in individuals with obesity [1,2]. AT is a tissue of mesenchymal origin, and it can be subdivided into white AT (WAT), brown and beige AT. AT is heterogeneous not only due to the existence of different AT types, but also because of its multi-depot distribution. Changes in the distribution of AT occur for a number of reasons, including declines in testosterone in men, and estrogen in women following menopause [3]. The ability of adipocytes to buffer dietary lipids declines with age, promoting the redistribution of lipids from subcutaneous to the abdominal visceral compartment [4]. These changes contribute to a low-grade state of inflammation, insulin resistance, and metabolic syndrome, contributing to an increased risk of type 2 diabetes, cardiovascular disease, and many other diseases associated with obesity. AT function and distribution during aging affects the synthesis of adipose tissue-derived mediators, or adipokines, and consequently regulates many physiologic processes, such as inflammation. Although WAT is distributed throughout the body, its principal deposits are in peripheral subcutaneous tissue (SAT) that is present on the hips and thighs, where it functions as an energy storage system, and the visceral or intra-abdominal region (VAT), where it protects against possible trauma. There is substantial evidence that accumulation of VAT shows higher angiogenesis [5,6] and a higher inflammatory profile than SAT, conferring a much higher cardiovascular risk to VAT than to SAT, which has shown a certain protective role against cardiometabolic disease [7,8,9]. Moreover, not all the VAT depots exhibit the same behavior in relation to obesity-associated inflammation and metabolic disturbances; three different VAT depots (mesenteric, omental, and periaortic), from patients with cardiovascular disease (CVD), have shown important differences in their inflammatory profile [10]. VAT and SAT show different adipokine expression profiles, distinct functions, morphologies, distributions, vascular density, and innervations [11,12], but both are composed by mature adipocytes and several other cells including preadipocytes, endothelial cells, and inflammatory cells, being considered as a large and accessible reservoir of mesenchymal stem cells, denoted as adipose-derived stem cells (ASCs) [13,14], which all together compose the stromal vascular fraction (SVF).

Similar to AT, ASCs from the two separate depots exhibit different characteristics [15], subcutaneous ASCs (SAT-ASCs) have a higher capacity to proliferate and to differentiate into an adipogenic lineage than visceral ASCs (VAT-ASCs) [16] and SAT-ASCs have highly expressed genes involved in transcription, contributing to proliferation, whereas VAT-ASCs have upregulated clusters of genes related to lipid biosynthesis and metabolism [17].

ASCs are currently the focus of interest in the field of inducible spontaneous regeneration and cell therapy [18,19]. Many groups have focused on ASCs-derived angiogenesis, and it is believed that their most relevant mechanism proceeds via paracrine actions, rather than endothelial differentiation of ASCs [20,21,22,23]. Apart from soluble growth factors, cell-released microvesicles (MVs)/exosomes have been recognized recently as a new mechanism of intercellular communication [24]. One of the mechanisms by which MVs from ASCs promote angiogenesis seems to be by delivery of miR-31 [25].

Even though there are some studies reporting on the different behavior of VAT and SAT and their role in angiogenesis [5,6] there is little agreement on the depot’s functional specificities. Recently we have demonstrated that resident ASCs in human epicardial AT display a depot-specific angiogenic function depending whether they are from ventricular myocardium AT or from the area covering the epicardial arterial sulcus of the left anterior descending artery [26]. Moreover, we have described that ASCs, in both humans and in rats, are functionally affected by cardiovascular risk factors (CVRFs) [22,27,28]. Particularly epicardial ASCs in animals with CVRFs lose their original angiogenic potential [29] or SAT-ASCs are committed to the adipogenic lineage, while their proliferation rate and proangiogenic potential are impaired in patients with obesity [22,30].

Here, our objective has been to investigate the angiogenic potency of human SAT and VAT in obese and lean patients. SAT and VAT were obtained from the same obese patients, and we studied not only the total fat tissues, but also their resident SAT-ASCs and VAT-ASCs. In addition, we investigated whether comorbidities (CVRFs), in addition to obesity, affected angiogenesis.

## 2. Materials and Methods

### 2.1. Patient Recruitment and Adipose Tissue Sampling

SAT and VAT were obtained via surgical resection from young individuals with morbid obesity (BMI  > 40 kg/m^2^; *n*  = 22) who underwent bypass gastric surgery. In these obese patients, AT was obtained simultaneously from subcutaneous and visceral depots during surgery. Additionally we collected adipose tissue from young individuals with normal weight (BMI < 25 kg/m^2^; *n*  = 15) who underwent abdominal lipectomy.

Informed consent was obtained from all donors and the study protocol was approved by the Centro Medico Teknon Ethical Committee that is consistent with the principles of the Declaration of Helsinki. Patients used regular medication as recommended in the guidelines, if it was necessary (Table 1).

### 2.2. RNA and cDNA Isolation from Adipose Tissue

Flash frozen adipose tissue was crushed using a mortar and pestle, and then RNA extraction was performed using a combination of organic extraction with Qiazol Reagent (Qiagen, Barcelona, Spain) and silica-membrane columns with the Qiagen RNeasy Mini Kit (Qiagen, Barcelona, Spain). RNA quantity was determined with a Nanodrop ND-1000 spectophotometer (Nanodrop Technologies, Wilmington, DE, USA). RNA quality was measured using 2100 Bioanalyzer technology (Agilent Technologies, Barcelona, Spain) with the Agilent RNA 6000 Nano Kit (Agilent Technologies, Barcelona, Spain) and assessed by the RNA integrity number (RIN). Only RNA samples with RIN values over 6 were chosen for real time PCR experiments.

First strand cDNA synthesis was performed using the high capacity cDNA reverse transcription kit (Applied Biosystems, Life Technologies, Madrid, Spain) from 500 ng of tissue extracted RNA according to manufacturer’s instructions.

### 2.3. Transcriptomic Analysis and RT-PCR

A custom gene expression low density array designed by Applied Biosystems (Life Technologies, Madrid, Spain) to characterize inflammation and angiogenesis was performed by quantitative PCR using TaqMan^®^ Gene Expression assays (Supplemental Table S1) using 200 ng of every sample following manufacturer’s instructions. TaqMan™ fluorescent real-time PCR primer used for tissue factor (TF) (Hs00175225_m1), VEGFA (Hs00900055_m1), miR126-3p (477887_miR), miR145-5p (477916_miR) and GAPDH (Hs99999905_m1), GUSB (Hs99999908_m1) and miR186-5p (477940_miR) (Applied Biosystems, Madrid, Spain) which were used as endogenous control. PCR data were analyzed with RQ Manager 1.2.1 and DataAssist 2.0 softwares (Applied Biosystems, Life Technologies, Madrid, Spain) against the endogens controls to obtain expression values for every gene (2-ΔCt).

### 2.4. Bioinformatic Analysis

The statistically significant neural network and the canonical pathway in which the studied genes were involved, were generated with IPA software (Ingenuity System, www.ingenuity.com). The functional analysis of a network was used to identify new target genes involved in the predicted pathways analyzed. The network molecules associated with biological functions and/or diseases in the Ingenuity Knowledge Base were considered for the analysis.

### 2.5. ASCs Isolation and Characterization

SAT and VAT were washed with sterile phosphate buffered saline (PBS) supplemented with 100 U/mL of penicillin and 100 μg/mL of streptomycin. Tissue was digested into a type I collagenase solution (1 mg/mL; Sigma-Aldrich, St. Louis, MO, USA) and incubated for 1 h in a 37 °C pre-warmed orbital shaker. Collagenase activity was neutralized with the same amount of fetal bovine serum (FBS; Biological Industries, Kibbutz Beit-Haemek, Israel) and the suspension filtered through a 100 μm mesh filter to eliminate remaining tissue fragments, then the solution was centrifuged at 1200 rpm for 10 min to separate the adipocytes and to obtain the stromal vascular fraction (SVF). Isolated SVF cells were counted and either analyzed by flow cytometry or plated onto a 25 cm^2^ culture flasks. After 24 h, non-adherent cells were removed and the medium replaced. Cells were expanded in a humidified environment at 37 °C with 5% CO_2_, and maintained at subconfluent levels prior to phenotypic profile analysis. The identity of ASCs was defined by using the following criteria: adherence to plastic, cell surface antigen phenotyping and differentiation into multiple cell lineages. All analyses were performed between passages 3 to 4.

For cell cytometry characterization, cell surface antigen phenotype was performed on ASCs of the SVF at passage 3 (P3). The following cell-surface epitopes were marked with anti-human antibodies: CD 105, CD 44, CD 29, CD 90, CD 73, CD 45 and CD 14 (Supplemental Table S2). 1 × 10^5^ cells at P3 or 1 mL of the SVF were suspended in flow cytometry buffer (PBS, 0.1% BSA, 0.1% sodium azide) and incubated for 30 min at 4 °C with the corresponding antibodies. After that, reaction was stopped by adding 500 µL of flow cytometry buffer or 250 µL of Quicklysis reagent (Cytognos, Salamanca, Spain) in the case of the SVF. Quicklysis was incubated for 15 min at room temperature to eliminate erythrocytes and reaction stopped by adding other 250 µL of flow cytometry buffer.

Cellular events (at least 30,000 in the case of P3 cells and between 10,000 and 60,000 in the case of the SVF) were acquired and analyzed by fluorescence-activated cell sorting using Coulter EPICS XL flow cytometer (Beckman Coulter, Barcelona, Spain) running Expo32 ADC XL 4 color software (Beckman Coulter, Barcelona, Spain).

### 2.6. MTS Viability/Proliferation Analysis

Cell proliferation was determined by 3-(4,5-dimethylthiazol-2-yl)- 5-(3-carboxymetho-xyphe-nyl)-2-(4-sulfopheny)-2H-tetrazolium (MTS) assay (CellTiter 96 Aqueous One Solution cell proliferation assay kit; Promega, Madison, WI, USA). For this assay, 15 × 10^3^ cells were seeded in triplicates into a 96-well plate, and 24 h later, 10 mL of MTS per well were added and then incubated for another 2 h while MTS tetrazolium was reduced to formazan (490 nm absorbance) by the metabolically active cells. The absorbance was then quantified. Formazan production was directly related with the number of cells alive in the culture.

### 2.7. Microvesicles Isolation

ASCs derived microvesicles (MVs) were isolated by ultracentrifugation of P3 cell supernatants as previously described [12]. Briefly, fresh supernatants were firstly centrifuged at 900× *g* for 15 min to eliminate cell debris and then at 20,000× *g* for 45 min to isolate the MVs as a pellet. MVs concentration was determined by flow cytometry. For that, MVs were washed with a PBS-citrate buffer and centrifuged again at 20,000× *g* for 30 min. MVs were extracted with PBS-citrate buffer and incubated with annexin V (CF Blue ANXV, Immunostep, Salamanca, Spain) and anti-TF antibody (FITC conjugated 4508CJ, Sekisui, Maidstone, UK). Samples were then diluted with annexin V binding buffer (BD Bioscience, Madrid, Spain) to stop the reaction and then analyzed on a FACSCantollTM flow cytometer (BD Bioscience, Madrid, Spain).

### 2.8. Cell Migration

For the cell migration assay, human microvascular endothelial cells (HMEC-1) (ATCC) were used. Briefly, 2.3 × 10^4^ ASCs were seeded into 100 mm dish and cultured with MCDB 131 medium supplemented with 10% of FBS for 48h to allow cells to secrete MVs. The day after, 2.5 × 10^5^ HMEC-1 cells were seeded into a culture-insert 2 well dish (Idibi) and kept with MCDB 131 supplemented with 10% of FBS overnight. MVs from the ASCs supernatant were isolated and before performing the experiment, the insert was removed by using sterile tweezers and the dish washed with PBS to remove cell debris. Cells were treated with 600 µL of: (A) Conditional medium from ASCs after 48 h of culture; (B) Conditional medium from ASCs without MVs after 48 h of culture; and (C) ASCs derived MVs rich medium. In all conditions the medium was supplemented with 2% of FBS. Cell migration and wound repair were controlled every 2 h for 12 h. Wound areas were analyzed by using Image J software. Protein, RNA and miRNA were isolated from the ASCs, miRNA from the MVs and RNA and miRNA from the HMEC-1 cells after 24 h of cell migration.

### 2.9. Gene Expression Analysis from ASCs

Total RNA was isolated from ASCs in silica-membrane columns with the Qiagen RNeasy Mini Kit (Qiagen, Barcelona, Spain) according to the manufacturer’s instructions.

MirVana miRNA isolation kit (Life Technologies, Madrid, Spain) was used to extract miRNA from the cells, and miRNeasy Serum/Plasma Kit for the miRNA isolation from MVs, according to the manufacturer’s instructions.

RNA and miRNA quantity was determined with Nanodrop ND-1000 spectophotometer (Nanodrop Technologies, Wilmington, DE, USA). Isolated total RNA was reverse-transcribed into cDNA using a high capacity cDNA archive kit (Applied Biosystems, Foster City, CA, USA), and microRNA with the TaqMAn advanced miRNA assay (Life Technologies, Madrid, Spain). Gene expression analysis was carried out by quantitative PCR using TaqMan^®^ Gene Expression assays (Applied Biosystems, Madrid, Spain), and the Applied Biosystems Prism 7900HT Sequence Detection System (Applied Biosystems, Madrid, Spain) according to manufacturer’s instructions. Gene expression data are expressed as target gene mRNA expression relative to the correspondent housekeeping gene expression.

### 2.10. Western Blot Analysis

Protein was extracted from total cell lysates by using RIPA buffer (50 mM Tris–HCl pH 8, 150 mM NaCl, 1% NP-40, 0.5% sodium Deoxycholate, 0.1% SDS) or from 48 h cell supernatant. Protein concentrations were measured with Pierce BCA Protein Assay Kit (ThermoScientific, Madrid, Spain). Twenty-five micrograms of protein were resolved by 1-DE under reducing conditions onto 10% SDS-PAGE gels and electrotransferred to nitrocellulose membranes. After blocking for non-specific binding, membranes were incubated with primary antibody; TF and beta-actin. Band detection was performed using a chemiluminiscent substrate dye (SuperSignal^®^ West Dura Extended Duration Substrate, Thermo Scientific, Waltham, MA, USA) and a molecular imager ChemiDoc XRS System, Universal Hood II (BioRad, Hercules, CA, USA). Band quantification was performed with Quantity-One software (BioRad laboratories, Hercules, CA, USA). Protein load was normalized with beta-actin.

### 2.11. Statistical Analysis

Data are expressed as mean and standard error unless stated. N indicates the number of subjects tested. Statistical analysis was performed with Stat View 5.0.1 software (Abacus Concept). The Kolmogrow–Smirnov test was performed to assess sample normality and then, non-parametric Willcoxon or Mann–Whitney analysis or parametric t-test analysis was performed depending on the compared samples and gene expression distribution. A *p*-value ≤ 0.05 was considered significant.

## 3. Results

### 3.1. Influence of Obesity on the Subcutaneous Adipose Tissue

**Figure 1:**
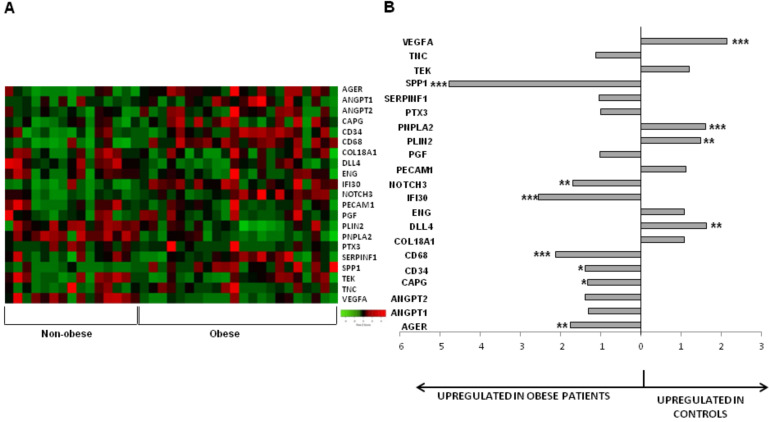
(**A**) Heat map of the gene expression in the subcutaneous fat of volunteers with normal weight versus patients with obesity; (**B**) Ratio of gene expression in subcutaneous fat of volunteers with normal weight versus patients with obesity. * *p* < 0.05; ** *p* > 0.01; and *** *p* < 0.001.

**Figure 2:**
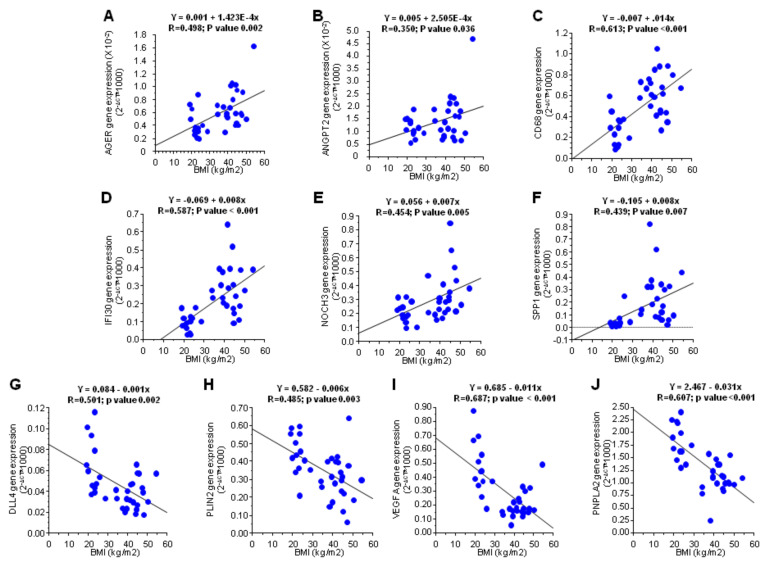
Line regression between expression of validated genes and body mass index (BMI). Correlations were determined by linear correlations. (**A**) Line regression between AGER and body mass index; (**B**) Line regression between ANGPT2 and body mass index; (**C**) Line regression between CD68 and body mass index; (**D**) Line regression between IFI30 and body mass index; (**E**) Line regression between NOCH3 and body mass index; (**F**) Line regression between SPP1 and body mass index; (**G**) Line regression between DLL4 and body mass index; (**H**) Line regression between PLIN2 and body mass index; (**I**) Line regression between VEGFA and body mass index; (**J**) Line regression between PINPLA2 and body mass index.

A custom gene expression assay was performed to assess the differential gene expression of 21 genes in SAT obtained from individuals with normal weight or obesity (Figure 1A; Supplemental Table S3). A significant up-regulation of genes involved in inflammation, immunity and cell proliferation was observed in the SAT of obese, compared to individuals with normal weight; AGER, CAPG, CD34, CD68, IFI30, NOTCH3 AND SPP1 (Table 2; Figure 1B). On the contrary, three genes involved in angiogenesis and one involved in energy homeostasis were up-regulated in thew SAT of individuals with normal weight compared to individuals with obesity; DLL4, PLIN2, VEGFA and PNPLA2. Additionally, a direct correlation between BMI and gene expression was observed for AGER, ANGPT2, CD68, IFI30, NOTCH3 and SPP1 (Figure 2A–F), whereas an inverse correlation was achieved for DLL4, PLIN, PNPLA2 and VEGF (genes that were down-regulated due to obesity) (Figure 2G–J). These results indicate that obesity decreases the angiogenic capacity of AT. 

Ingenuity pathway analysis (IPA^®^) data were used to identify new targets of the networks affected by these changes. The main affected network was related to the “cardiovascular system development and function, tissue and cell morphology”, with a score of 38 and 16 of our studied genes involved (Supplementary Figure S1A).

### 3.2. Influence of Obesity in Fat Depot Genomic Profile

**Figure 3:**
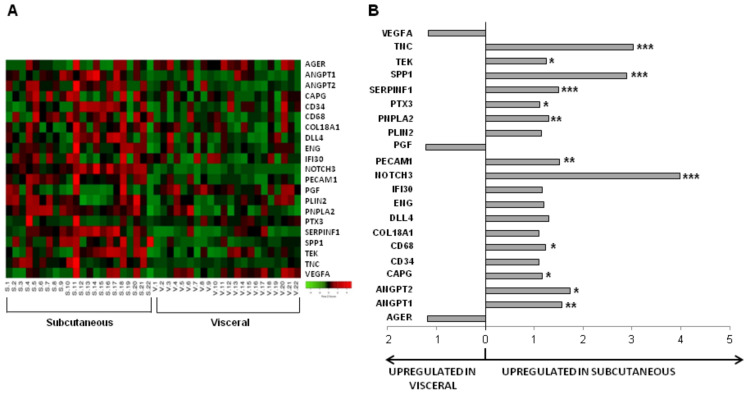
(**A**) Heat map of the gene expression in subcutaneous versus visceral fat; (**B**) Fold change of gene expression in subcutaneous versus visceral fat. * *p* < 0.05; ** *p* > 0.01; and *** *p* < 0.001.

Gene expression was investigated in SAT and VAT (Figure 3A) and their derived ASCs (S-ASCs and V-ASCs, respectively), obtained simultaneously from patients with obesity during bariatric surgery. SAT as compared to VAT showed higher transcript levels of genes involved in inflammation and angiogenesis (ANGPT1, ANGPT2, CAPG, CD68, NOTCH3, PECAM1, PTX3, SERPINF1, SPP1, TEK and TNC) as well as in PNPLA2, which is involved in energy homeostasis (Figure 3B and Table 3 column 3). In contrast, AGER, PGF and VEGFA were non-significantly upregulated in VAT compared with SAT. AT depots exhibit different angiogenic potential, with SAT showing higher angiogenic gene transcription levels than VAT.

To elucidate whether obesity was the main cause of these changes, or on the contrary other CVRFs were involved, we analyzed the data, excluding patients with obesity without CVRFs (only patients with obesity were excluded from the analysis) and evidenced that most of the gene changes previously seen between SAT and VAT remained within the same trend as previously observed. This was true for all, except for PTX3, which lost the significance, and IFI30, which reached a significantly higher expression in SAT compared with VAT (Table 3 column 4), indicating that changes in IFI30 may be related to obesity.

Next, we analyzed the effect of each CVRFs independently. First, we compared expression levels in SAT and VAT from obese/non-diabetic and obese/diabetic subjects. When non-diabetic patients with obesity were analyzed, eleven genes were significantly modified; all of them overexpressed in SAT (ANGPT1, ANGPT2, CD68, DLL4, NOTCH3, PECAM1, PNPLA2, PTX3, SERPINF1, SPP1 and TNC) (Table 3 column 5). However, comparative analysis of SAT and VAT from diabetic patients with obesity showed that only one gene was significantly higher expressed in SAT compared to VAT, NOTCH3 (Table 3 column 6). No differences were found when we analyzed the effects of DM in SAT (non-DM versus DM) or in VAT (non-DM versus DM) (Supplemental Table S4). Therefore, DM induces a different transcriptomic profile in SAT versus VAT.

When we studied the effect of dyslipidemia (DLP), only one gene was significantly reduced in SAT, COL18A1. No changes regarding DLP were observed in VAT (Supplemental Table S5). We also analyzed the different behavior of SAT and VAT from obese/non-DLP patients and we found eight genes significantly overexpressed in SAT as compared to VAT; ANGPT1, CAPG, NOTCH3, PNPLA2, SERPINF1, SPP1, TNC and VEGFA, and only one gene was found to be downregulated, PTX3 (Table 3 column 7). On the other hand, when we compared SAT and VAT from obese DLP patients, we observed that ten genes were more highly expressed in SAT as compared to VAT; ANGPT1, ANGPT2, COL18A1, DLL4, NOTCH3, PECAM1, PNPLA2, SERPINF1, SPP1 and TNC (Table 3 column 8).

Finally, we investigated the effects of arterial hypertension (HT) on the angiogenic potential of both SAT and VAT, and we observed a significantly higher level of expression of ANGPT1 and a significantly reduced level of expression of TNC in the SAT of obese HT patients with obesity as compared to nHT patients with obesity (Supplemental Table S6). When we specifically analyzed nHT patients with obesity we observed that VEGFA was significantly reduced in SAT. In addition, another eight genes showed a higher level of expression in SAT as compared to VAT; ANGPT1, ANGPT2, DLL4, NOTCH3, PECAM1, SERPINF1, SPP1 AND TNC (Table 3 column 9). We also analyzed the genomic changes between SAT and VAT of HT patients, finding a higher level of expression of five genes in SAT as compared to VAT; IFI30, NOTCH3, PNPLA2, SERPINF1, and TNC (Table 3 column 10).

We further analyzed the networks in which these genes were involved using the IPA software analysis in order to investigate new potential targets associated with the observed changes. We found that, as expected, the “cardiovascular system development and function, tissue and cell morphology” network was affected, also with a score of 38 and 16 molecules involved (Supplemental Figure S1B).

### 3.3. Angiogenic Related Gene Expression in Subcutaneous or Visceral Derived ASCs from Diabetic/No Diabetic Patients with Obesity

**Figure 4:**
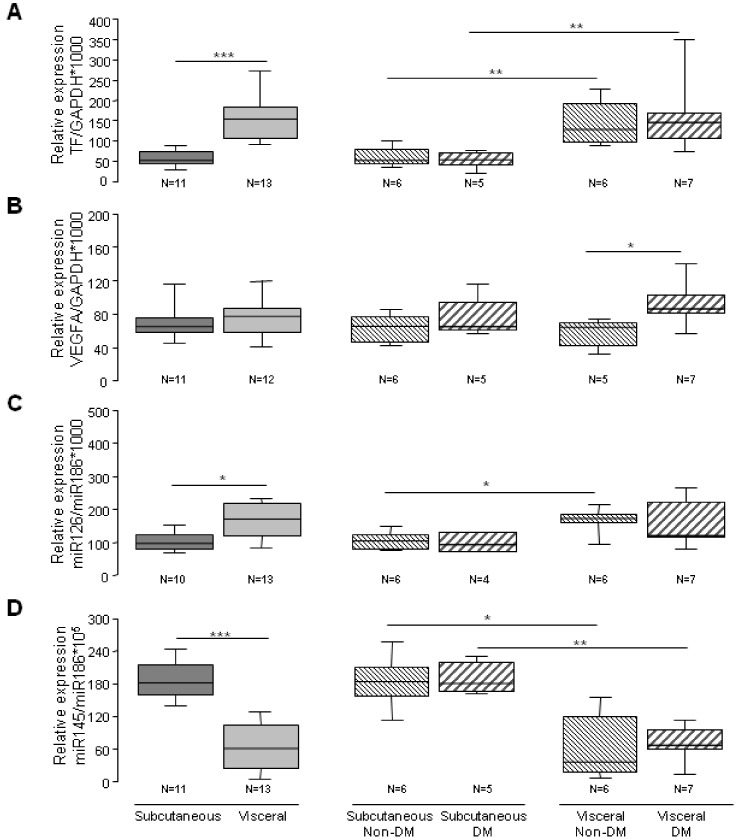
Box-plot diagram showing adipose-derived stem cells (ASCs)-transcriptomic profile. (**A**) Differential mRNA expression of TF in SAT-ASC and VAT-ASC from diabetic with obesity and non-diabetic patients; (**B**) Differential mRNA expression of VEGFA in SAT-ASC and VAT-ASC from obese diabetic and non-diabetic patients; (**C**) Differential miR126 expression in SAT-ASC and VAT-ASC from obese diabetic and non-diabetic patients; (**D**) Differential miR145 expression in SAT-ASC and VAT-ASC from diabetic with obesity and non-diabetic patients. (* *p* < 0.05; ** *p* > 0.01; and *** *p* < 0.001).

**Figure 5:**
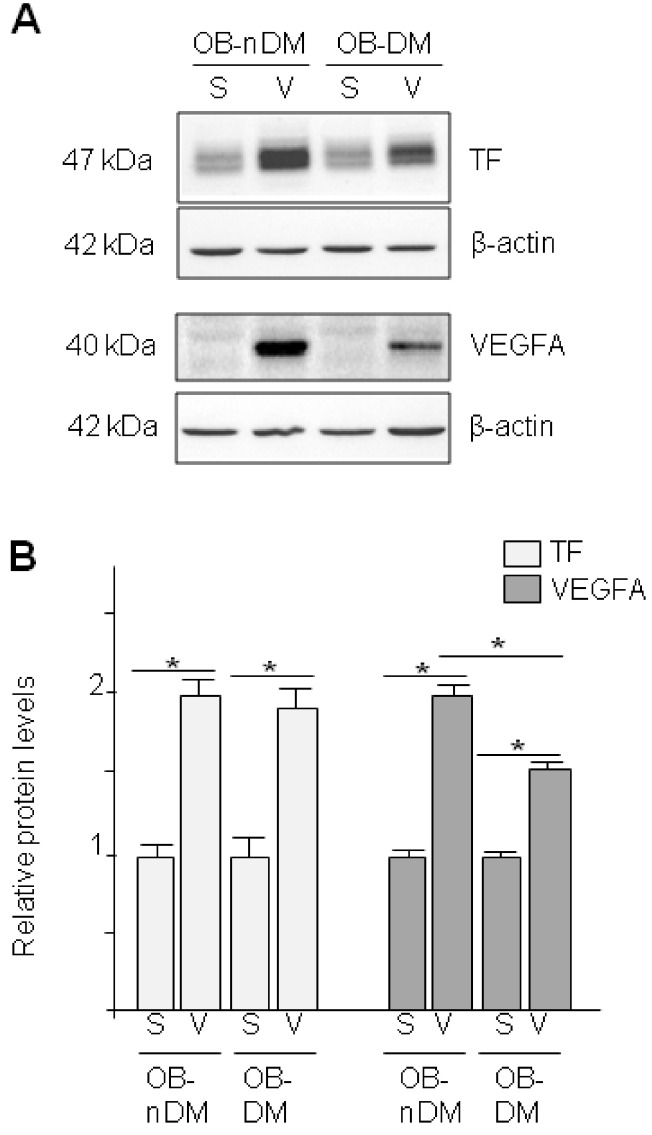
TF and VEGF expression in in Subcutaneous or Visceral Derived ASCs from Diabetic/No Diabetic Patients with Obesity. (**A**) TF and VEGFA expression in ASCs obtained from SAT (S) or VAT (V) in obese patients (OB) in presence (DM) or absence (nDM) of diabetes. Western blot analysis of TF and VEGFA proteins in ASCs. To test for equal loading Western blots were reproved by β-actin; (**B**) Quantitative analysis of TF and VEGFA to β-actin relative levels.

ASCs have been proposed as pro-angiogenic therapy in diabetic patients for treating ischemic complications. We analyzed whether obesity and diabetes affected molecules involved in angiogenesis; such as TF, VEGFA and two miRNAs (miR-126 and miR145) [31,32]. First, we investigated the angiogenic potential of SAT-ASCs of subjects with normal weight or obesity and we found no significant changes on the analyzed genes due to obesity (Supplemental Figure S2). Next, we studied the effect of DM and fat depot (SAT-ASCs versus VAT-ASCs) on the same molecules. The comparison of the differential expression yielded a significant up-regulation of the proangiogenic gene/protein TF (*p* < 0.001; Figure 4A and Figure 5A) and microRNA miR126-3p (*p* = 0.015; Figure 4C) in VAT-ASCs, whereas miR145 was down-regulated (*p* < 0.001; Figure 4D). There was a significant increase in VEGFA gene expression in VAT-ASCs from diabetic patients compared with non-diabetics (*p* = 0.019; Figure 4B). However, when we analyzed miR145 (an antiangiogenic miRNA), we observed that it was reduced in VAT-ASCs as compared to SAT-ASCs and no differences were observed due to the presence of diabetes.

Interestingly, TF protein was significantly higher in VAT-ASC than SAT-ASCs independently of DM, while VEGFA protein levels (Figure 5A) were higher in VAT-ASCs than SAT-ASCs, but there was a significant effect of DM. Indeed, protein VEGFA levels were lower in VAT-ASCs from diabetic patients as compared to VAT-ASCs from non-diabetic patients.

### 3.4. Subcutaneous and Visceral ASCs Derived Microvesicles and Effects on Wound Repair

**Figure 6:**
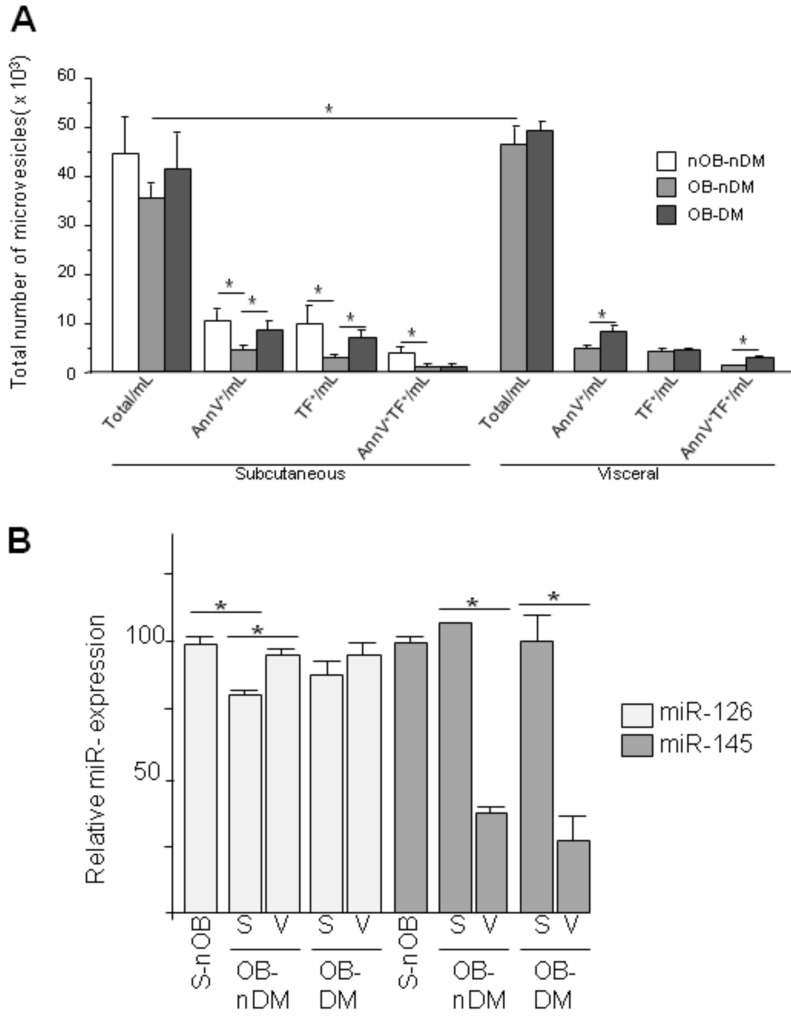
(**A**) ASC microvesicles (MVs) secretion. Influence of obesity and diabetes on ASC derived MVs. Number of MVs measured by flow cytometry (Total, annexin-V positive, TF positive and annexin-V/TF positive MVs); (**B**) miR126 and miR145 relative gene expression in S-MVs and V-MVs. (* *p* < 0.05).

The angiogenic potential of ASCs had been attributed to exocrine functions, particularly microvesicles (MVs) released from ASCs [33]. Given that, TF may be secreted in form of MVs, the number of MVs released by ASCs, from each fat depot in non-obese/obese and non-diabetic/diabetic individuals, was measured by flow cytometry. No differences were found in the number of total MVs released by the subcutaneous derived ASCs (S-MVs) from non-obese or obese individual (Figure 6A). Significant differences (*p* = 0.015) were found in S-MVs as compared to visceral derived ASCs MVs (V-MVs), visceral ASCs released significantly more MVs than subcutaneous ASCs. Moreover, results show that annexin V+, TF + or both positive MVs released by subcutaneous ASCs were decreased in individuals with obesity as compared to individuals with normal weight, no differences were observed between S-MVs or V-MVs. However, DM reversed the effect induced by obesity. When we separated groups by adipose tissue location, we found that this statistical significance remained.

Since released MVs carry miRNA from the parental cells, we analyzed miR-126 and miR-145 in MVs. The results showed that expression of miR-126 was significantly reduced in MVs obtained from obese-ASCs as compared to non-obese patients, and expression levels were restored in V-ASCs (Figure 6B). Diabetes did not modify the expression of miR-126 in MVs neither from SAT-ASCs or VAT-ASCs. In contrast, miR-145 expression was significantly higher in MVs obtained from S-ASCs than those obtained from VAT-ASCs both in non-diabetic and diabetic patients.

**Figure 7:**
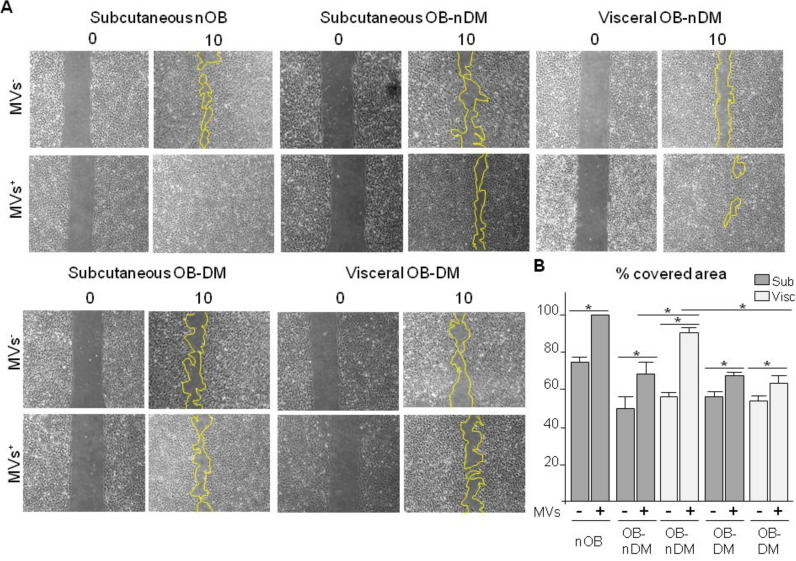
Effect of ASC-MVs in endothelial cell migration. Human microvascular endothelial (HMEC-1) cells treated with ASC conditioned medium, MVs depleted or MVs rich medium. (**A**) Images show the wound repair at 10 h; (**B**) Cell coverage of the wounded area at 10 h. Results are expressed as % of covered wounded area ± SEM. * *p* < 0.05.

Next, in order to analyze the induced-migration potential of MVs released by ASCs as paracrine mediators, a migration assay with human microvascular endothelial cells (HMEC-1) was performed. Confluent HMEC-1s cultures were wounded by a fixed size scratch and treated with MVs released by ASCs to investigate the repair potential. After scratching, HMEC-1 were cultured with conditioned media (CM) obtained after 48 h of ASCs culture. CM was centrifuge to obtain MVs (MVs+) and CM without MVs (MVs-). Wound healing was quantified by measuring the covered surface 10 h after the scratch (time needed to close the gap) (Figure 7). Wounds closed faster in HMEC-1 treated with CM rich MVs (MVs+) than those cells treated with CM poor MVs (MVs-). Moreover, results show that MVs from VAT-ASCs have a higher capacity to induce migration than MVs from SAT-ASCs. Additionally, the presence of diabetes reduces migration capacity. All results were corrected by the number of MVs.

**Figure 8:**
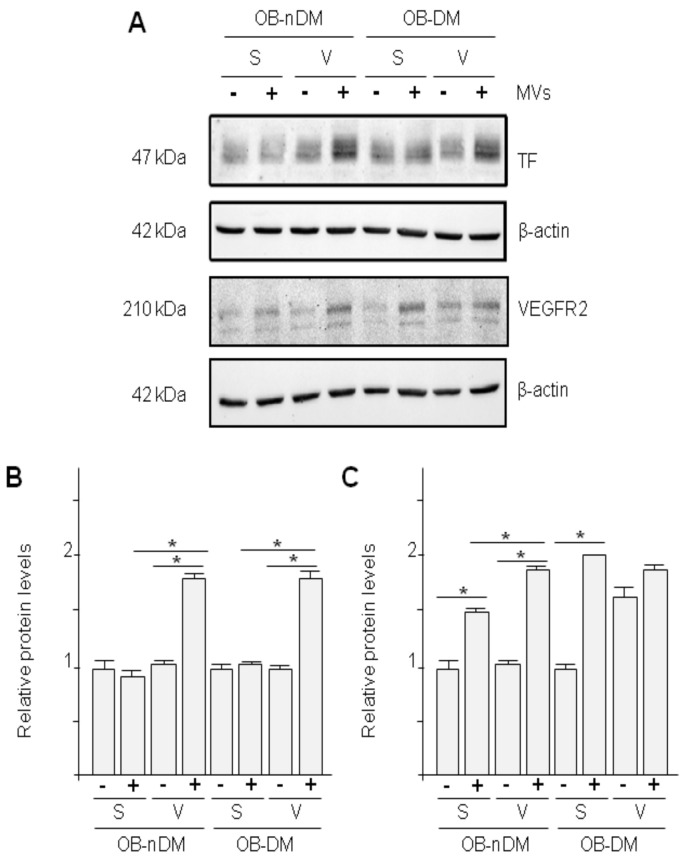
Effect of ASCs-MVs on protein expression in protein endothelial cells. (**A**) TF and VEGFR2 protein expression in migrating endothelial cells treated with media with (+) or without (-) MVs obtained from 48 h cultured of subcutaneous (S) or visceral (V) ASCs from patients with obesity without diabetes (OB-nDM) or with diabetes (OB-DM); (**B**) Quantitative analysis of TF to β-actin relative levels; (**C**) Quantitative analysis of VEGFR2 to β-actin relative levels. (* *p* < 0.05).

Because we observed changes in MVs content and in scratch-time closing in HMEC-1 treated with MVs from ASCs, we analyzed TF and VEGFR2 (receptor for VEGFA). MVs from VAT-ASCs induced significantly higher TF and VEGFR2 expression in HMEC-1 cells (Figure 8).

## 4. Discussion

Over the past decades, AT perception has changed considerably because of the dramatic increase in the incidence of obesity and obesity-related comorbidities [34]. Moreover, AT has also been considered as a source of ASCs for organ regeneration therapies due to their angiogenic properties [35]. The growing of AT requires the support of the angiogenic process [3] and it is known that AT from different anatomical localizations have different angiogenic capacities [24]. Our hypothesis is that adipose tissue, depending on its location, as well as its derived ASCs, presents different angiogenic capacity. This ability is also modified not only by the presence of obesity but also by other risk factors. In our study, we have used a transcriptomic approach to investigate the effect of different CVRFs, individually or as a cluster, on the angiogenic and inflammatory behavior of AT from two different depots in the same patient, the subcutaneous and visceral WATs. We analyzed 21 genes involved in different angiogenic or inflammatory pathways. Additionally, to investigate the behavior of fat tissue and its resident stem cells, ASCs from these fat depots were also isolated.

In SAT from obese patients, a significant reduction in the VEGFA expression and an increase in serpin family F member 1 (SERPINF1, also known as pigment epithelium-derived factor, PEDF) expression was observed with respect to SAT obtained from lean healthy donors. Since PEDF can inhibit VEGF-mediated angiogenesis in the endothelial cells (EC) by activating ɣ-secretase, which cleaves VEGFR after the translocation of the C-terminal region of the receptor [36,37,38], a significant inhibition of angiogenesis ensues in obese SAT. However, no differences were observed in VEGFA gene expression in the SAT-ASCs from both obese and non-obese subjects, suggesting than the angiogenic potential of SAT could be related with the non-mesenchymal cell components of the tissue. Contrary to what we could expect from previous studies [39,40,41], the levels of the PNPLA2, gene involved in the maintenance and development of AT, as well as in regulating energy homeostasis, was significantly lower in the SAT of patients with obesity as compared to individuals with normal weight. Schrammel et al. [42] have demonstrated that ATGL knockout mice (ATGL is an enzyme that in humans is encoded by the *PNPLA2* gene) suffer a pronounced micro and macrovascular endothelial dysfunction and activation of lipolysis by exercise modified angiogenic gene expression in a fat depot specific manner [43].

It is accepted that SAT and VAT have different compositions and different metabolic influence and thus, each depot has a different transcriptome and proteome [44,45,46]. Although obesity is widely known as a risk factor for different pathologies, many studies have shown that being overweight and obesity can exert, in certain cases, a protective role, giving rise to what is known as the “paradox of the obesity”. Thus, SAT is a protective tissue, while VAT is much more damaging [3,4]. In fact, it is demonstrated that insulin resistance is associated with VAT but not with SAT. Obesity and fat accumulation can be related with two different processes related to adipocytes, hypertrophy (enlarged adipocytes) or hyperplasia (increased number of adipocytes), the first being the predominant contributor to adult obesity. Conversely, metabolic healthy obesity is associated with an increased number of small adipocytes, and therefore with adipocyte hyperplasia. We show that human SAT and VAT in obese and lean patients, not only in the total fat tissues but also their resident SAT-ASCs and VAT-ASCs, have different angiogenic properties.

When we analysed the transcriptomic profile of SAT and VAT fat, we observed a higher pro-angiogenic gene expression in SAT compared with VAT of the same obese patients, as for the 21 genes analyzed, only three had a higher expression in VAT. Thus, the observed angiogenic-related gene expression increase observed in the SAT compared with VAT suggests that adipocytes hyperplasia predominates in SAT, which as previously discussed is predominant in women.

**Figure 9:**
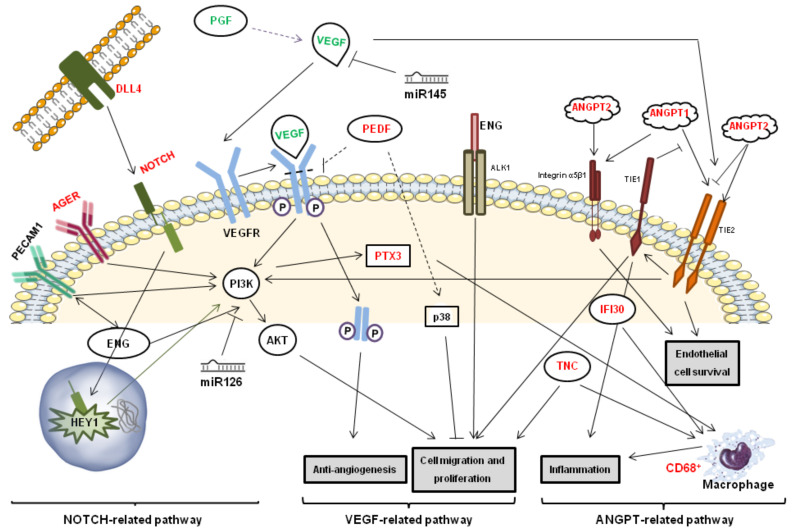
Inflammatory and angiogenic pathways differentially affected in SAT versus VAT, in green downregulated and red up regulated.

AT is composed of adipocytes and preadipocytes, stromal cells, macrophages and leukocytes among others, which secrete inflammatory and angiogenic factors, therefore being central mediators of the angiogenic process [47]. Here, we have observed that in obese patients, different angiogenic pathways are activated depending on the AT depots analyzed. We have evidenced that the VEGF-mediated angiogenic pathway, which involves genes such as PGF, VEGFA and PEDF, was partially blocked in SAT. However, other pathways such as the ANGPT and NOTCH pathways among others, were enhanced (Figure 9). Additionally in SAT compared with VAT, there was a significant overexpression of ANGPT1 and ANGPT2, as well as their receptor TIE2. This ANGPT-TIE2 system, gives a connection between the angiogenic and the inflammatory pathways [48]. ANGPT1 induced TIE-2 phosphorylation (p-Tie-2), which leads to the maintenance of EC stability. The role of ANGPT1 and VEGFA on blood vessel growth seems to be complementary, since both genes can induce TIE-2 phosphorylation and therefore maintain endothelial homeostasis [49]. On the contrary, ANGPT2 does not induce p-Tie-2 and therefore is considered as an ANGPT1 antagonist. However, ANGPT2, which is mainly produced by EC, promotes angiogenesis by promoting the dissociation of pericytes from old vessels and facilitating the liberation of diverse pro-angiogenic and pro-inflammatory molecules [47,48,49,50]. In addition to the observed effects on the ANGPT-TIE2 system, we also observed increased expression of NOTCH3 and its ligand DLL4 on SAT compared with VAT. The Notch signaling pathway is composed of four receptors (Notch1–4) and five membrane-bound ligands (Jag1-2 and DLL1,3 and 4) [51]. When these ligands interact with any of the receptors, TNFα and **ɣ**-secretase induce the cleavage of the intracellular NOTCH-domain, which translocates to the nucleus, where it activates expression of target genes responsible for different cellular decisions and functions. NOTCH activation enhanced the phosphatidylinositol 3-kinase (PI3K)/AKT pathway promoting cell migration and proliferation and therefore, promoting angiogenesis [52,53,54,55] (Figure 9).

Besides the differential regulation of these three pathways between the different AT depots, other genes were also differentially expressed. Platelet endothelial cell adhesion molecule-1 (PECAM1), endoglin (ENG) and advanced glycation end-products (AGER) were more highly expressed in the SAT compared to the VAT. These three genes have an important role in the PI3K/AKT cell migration and proliferation related pathways. In fact, ENG and PECAM1 have a great influence over one another, and PECAM1 deficiently is associated with ENG reduction [56,57]. capping actin protein (CAPG) is also more highly expressed in SAT than in VAT. CAPG is a member of the gelsolin family, with an important role in endothelial and fibroblast cell motility as well as in macrophage conservation [58,59]. The inflammatory pathway was also differentially regulated between both tissues, higher levels of CD68, a monocyte and macrophage surface marker, were observed in SAT compared with VAT, as well as increased expression of the interferon-gamma-inducible protein 30 (IFI30), expressed by macrophages [60] and tenascin-C (TNC), protein with roles both in inflammation and in cell migration and proliferation [61,62]. It is important to point out that all these differences disappeared if we only study the obese-diabetic group, due to the fact that SAT from patients with obesity might have a protective angiogenic role, whereas when obesity is accompanied by other metabolic diseases, SAT could be as deleterious a tissue as VAT.

Regarding the mesenchymal stem cell reservoir in these adipose tissues, we observed no significant changes in VEGFA expression, up-regulation in TF (F3) and miR-126 expression and down-regulation in miR-145 expression in the VAT-ASCs with regards to SAT-ASCs. miR-126 inhibits PIK3R2 and SPRED1, genes that in turn inhibit VEGFA [32], on the contrary, miR-145 has VEGFA as a direct target [63]. However, PIK3 is required for Tie-2 activation by ANGPT1, Tie-2 and ANGPT1 are therefore negatively regulated by miR-126 levels [64]. These results support the up-regulation of VEGFA, seen in the total visceral fat analysis, as well as the ANGPT1 and TIE-2 reduction. However, unlike what we saw in the total fat tissue, these changes are not affected by the presence of diabetes mellitus.

ASCs secrete MVs that may be involved in crosstalk functioning with target cells [65]. In our study we found improved wound healing and viability in the endothelial cells treated with S-ASCs derived MVs. However, this repair function was not VEGF-dependent.

Unfortunately, the main limitation of this study is the difficulty to recruit samples of these types of patients. However, besides this technical limitation, the changes observed are significant enough to help improve the existing knowledge in this field.

AT can be largely distributed around the human body, and therefore, its properties and composition differ from one location to the other. In this study we have analyzed, for the first time, the angiogenic potential of the subcutaneous and visceral adipose tissue and their derived stem cells, as well as their extracellular MVs, all obtained from the same subject. In this approach we minimized genetic differences in the comparisons. In addition to obesity, other comorbidities, were investigated. Even though more studies in this field are needed, we can conclude that adipose tissue is a very active organ, independent of location. The subcutaneous adipose tissue from obese patients, expresses a greater number of angiogenic molecules than the visceral fat, independent of the presence of other CVRFs. However, the VEGF pathway is partially blocked in SAT, both compared with the visceral adipose tissue and with the subcutaneous adipose tissue from lean healthy donors. We have also observed that, even though adipose tissue composition is very heterogeneous, the adipose stem cells and their derived microvesicles have a pivotal role in the angiogenic properties of the adipose tissue.

## Figures and Tables

**Table 1:** Clinical characteristics of the study patients.

Variables	nOB	OB
N	15	22
Age (Years)	40 ± 4	39 ± 2
Sex (M/W)	2/13	3/19
BMI (Kg/m^2^)	23.9 ± 1.1	43.0 ± 1.1
CVRF (N)	0	3
Smoking (%)	0	55
HT (%)	0	32
DM (%)	7	23
DLP (%)	0	50
RBC (×10^6^/mm^3^)	3.8 ± 0.4	3.9 ± 0.7
MCB (µm^3^)	81.7 ± 4.6	80.3 ± 5.03
RDV (%)	14.8 ± 1	15.7 ± 1.8
HCT (%)	30.8 ± 2.7	31.05 ± 5.6
PLT × 10^3^/mm^3^)	211 ± 47.4	182.7 ± 56.8
MPV (µm^3^)	9.1 ± 0.8	8.9 ± 1.1
WBC (×10^3^/mm^3^)	4.5 ± 0.4	4.9 ± 0.9
HGB (g/dl)	10.7 ± 1	11.06 ± 2.2
MCH (pg)	28.5 ± 1.4	29.03 ± 2.3
MCHC (g/dl)	34.9 ± 1.2	36.15 ± 2.2
LYMF (×10^3^/mm^3^)	1.3 ± 0.3	1.23 ± 0.5
GRAN (×10^3^/mm^3^)	2.6 ± 0.4	3.2 ± 0.3
MID (×10^3^/mm^3^)	0.5 ± 0.05	0.6 ± 0.08

nOB, normal weight. OB, Obese. CVRF, cardiovascular risk factors. HTA, hypertension. DM, Diabetes Mellitus. DLP, Dyslipidemia. Values are expressed as mean ± SD or as percentages, when indicated.

**Table 2:** Differential transcriptomic expression between subcutaneous adipose tissue (SAT) of individuals with normal weight and individuals with obesity.

	*p* Value	Ratio
AGER	0.001	1.75
ANGPT1	0.134	1.31
ANGPT2	0.094	1.39
CAPG	0.029	1.33
CD34	0.041	1.39
CD68	<0.001	2.13
COL18A1	0.417	0.93
DLL4	0.001	0.62
ENG	0.230	0.93
IFI30	<0.001	2.55
NOTCH3	0.005	1.70
PECAM1	0.416	0.88
PGF	0.944	1.02
PLIN2	0.002	0.67
PNPLA2	<0.001	0.62
PTX3	0.780	1.00
SERPINF1	0.939	1.04
SPP1	<0.001	4.76
TEK	0.104	0.83
TNC	0.183	1.12
VEGFA	<0.001	0.46

Red: up-regulated, green: down-regulated in patients with obesity *p* < 0.05.

**Table 3:** Differential genomic expression in SAT versus the visceral adipose tissue (VAT) of patients with obesity with different number of CVRFs.

Gene	Ratio	All Patients N = 22	cCRFs N = 19	Diabetes mellitus		Dyslipidemia		Hypertension	
				OB-nDM N = 17	OB-DM N = 5	OB-nDLP N = 11	OB-DLP N = 11	OB-nHT N = 15	OB-HT N = 7
AGER	0.85	0.072	0.184	0.193	0.138	0.051	0.534	0.140	0.176
ANGPT1	1.56	0.001	0.005	0.002	0.500	0.016	0.026	0.003	0.311
ANGPT2	1.73	0.011	0.009	0.013	0.500	0.374	0.013	0.027	0.176
CAPG	1.17	0.049	0.045	0.108	0.225	0.049	0.373	0.270	0.066
CD34	1.09	0.406	0.414	0.069	0.225	0.819	0.371	0.900	0.264
CD68	1.23	0.013	0.013	0.028	0.225	0.182	0.033	0.054	0.091
COL18A1	1.10	0.170	0.089	0.286	0.343	0.277	0.024	0.775	0.086
DLL4	1.30	0.053	0.070	0.042	0.893	0.497	0.029	0.016	0.828
ENG	1.20	0.055	0.081	0.087	0.345	0.289	0.110	0.105	0.243
IFI30	1.17	0.083	0.020	0.269	0.080	0.746	0.052	0.566	0.033
NOTCH3	3.98	<0.001	<0.001	<0.001	0.043	0.003	0.003	<0.001	0.018
PECAM1	1.52	0.002	0.006	0.006	0.225	0.062	0.016	0.004	0.176
PGF	0.83	0.072	0.091	0.084	0.686	0.051	0.594	0.100	0.866
PLIN2	1.15	0.143	0.382	0.280	0.686	0.073	0.825	0.226	0.428
PNPLA2	1.31	0.004	0.011	0.013	0.138	0.041	0.021	0.202	0.018
PTX3	1.11	0.046	0.108	0.050	0.500	0.013	0.657	0.088	0.237
SERPINF1	1.50	<0.001	<0.0001	<0.001	0.080	<0.001	0.004	0.002	0.018
SPP1	2.90	<0.001	0.003	0.003	0.225	0.021	0.016	0.001	0.237
TEK	1.24	0.029	0.031	0.050	0.500	0.213	0.086	0.128	0.145
TNC	3.03	<0.001	<0.001	<0.001	0.080	0.003	0.008	0.004	0.018
VEGFA	0.86	0.076	0.096	0.376	0.080	0.024	0.395	0.005	0.957

Red: upregulated in subcutaneous *p* < 0.005; green: downregulated in subcutaneous *p* < 0.05.

## Supplementary Materials

The following are available online at www.mdpi.com/xxx/s1.Table S1: TaqMan gene expression assay probes used in the custom array; Supplemental Table S2: Antibodies used to perform the experiments; Table S3: Gene expression profile of the subcutaneous and visceral adipose tissue of patients and controls; Table S4: Gene expression of SAT and VAT from non-diabetic (non-DM) and diabetic (DM) obese patients. Significance between non-DM versus DM; Table S5: Gene expression of SAT and VAT from non dyslipemic (non-DLP) and dyslipemic (DLP) obese patients. Significance between non-DLP versus DLP; Table S6: Gene expression of SAT and VAT from non hypertens (non-HP) and hypertens (HP) obese patients. Significance between non-HP versus HP; Figure S1: IPA network analysis; Figure S2: Box-plot diagram showing ASC genomic profile.

## Abbreviations

ASCs	Adipose derived stem cells
AT	Adipose tissue
CVRFs	Cardiovascular risk factors
DLP	Dyslipidemia
DM	Diabetes mellitus
HT	Hypertension
MVs	Microvesicles
OB	Obesity
S-	Subcutaneous
SAT	Subcutaneous adipose tissue
SAT-ASCs	Adipose derived stem cells obtained from subcutaneous adipose tissue
S-MVs	Microvesicles released by adipose derived stem cells obtained from subcutaneous adipose tissue
V-	Visceral
VAT	Visceral adipose tissue
VAT-ASCs	Adipose derived stem cells obtained from visceral adipose tissue
V-MVs	Microvesicles released by adipose derived stem cells obtained from visceral adipose tissue

## References

[1] Ailhaud G., Grimaldi P., Negrel R. (1992). Cellular and molecular aspects of adipose tissue development. Annu. Rev. Nutr..

[2] Smitka K., Maresova D. (2015). Adipose Tissue as an Endocrine Organ: An Update on Pro-inflammatory and Anti-inflammatory Microenvironment. Prague Med. Rep..

[3] Ley C., Lees J.B., Stevenson J.C. (1992). Sex- and menopause-associated changes in body-fat distribution. Am. J. Clin. Nutr..

[4] Karpe F., Pinnick K.E. (2015). Biology of upper-body and lower-body adipose tissue—Link to whole-body phenotypes. Nat. Rev. Endocrinol..

[5] Ledoux S., Queguiner I., Msika S., Calderari S., Rufat P., Gasc J.M., Corvol P., Larger E. (2008). Angiogenesis associated with visceral and subcutaneous adipose tissue in severe human obesity. Diabetes.

[6] Silverman K.J., Lund D.P., Zetter B.R., Lainey L.L., Shahood J.A., Freiman D.G., Folkman J., Barger A.C. (1988). Angiogenic activity of adipose tissue. Biochem. Biophys. Res. Commun..

[7] Fantuzzi G., Mazzone T. (2007). Adipose tissue and atherosclerosis: Exploring the connection. Arterioscler. Thromb. Vasc. Biol..

[8] Larsson B., Svärdsudd K., Welin L., Wilhelmsen L., Björntorp P., Tibblin G. (1984). Abdominal adipose tissue distribution, obesity, and risk of cardiovascular disease and death: 13 year follow up of participants in the study of men born in 1913. Br. Med. J. Clin. Res. Ed..

[9] Okura T., Nakata Y., Yamabuki K., Tanaka K. (2004). Regional body composition changes exhibit opposing effects on coronary heart disease risk factors. Arterioscler. Thromb. Vasc. Biol..

[10] Kranendonk M.E., van Herwaarden J.A., Stupkova T., de Jager W., Vink K., Moll F.L., Kalkhoven E., Visseren F.L.J. (2015). Inflammatory characteristics of distinct abdominal adipose tissue depots relate differently to metabolic risk factors for cardiovascular disease: Distinct fat depots and vascular risk factors. Atherosclerosis.

[11] Gil A., Olza J., Gil-Campos M., Gomez-Llorente C., Aguilera C.M. (2011). Is adipose tissue metabolically different at different sites?. Int. J. Pediatr. Obes..

[12] Ibrahim M.M. (2010). Subcutaneous and visceral adipose tissue: Structural and functional differences. Obes. Rev..

[13] Esteve Rafols M. (2014). Adipose tissue: Cell heterogeneity and functional diversity. Endocrinol. Nutr..

[14] Zuk P.A., Zhu M., Ashjian P., De Ugarte D.A., Huang J.I., Mizuno H., Alfonso Z.C., Fraser J.K., Benhaim P., Hedrick M.H. (2002). Human adipose tissue is a source of multipotent stem cells. Mol. Biol. Cell.

[15] Sadie-Van Gijsen H., Crowther N.J., Hough F.S., Ferris W.F. (2010). Depot-specific differences in the insulin response of adipose-derived stromal cells. Mol. Cell Endocrinol..

[16] Toyoda M., Matsubara Y., Lin K., Sugimachi K., Furue M. (2009). Characterization and comparison of adipose tissue-derived cells from human subcutaneous and omental adipose tissues. Cell Biochem. Funct..

[17] Kim B., Lee B., Kim M.K., Gong S.P., Park H.Y., Chung Y.Y., Kim H.S., No J.H., Park W.Y., Park A.K. (2016). Gene expression profiles of human subcutaneous and visceral adipose-derived stem cells. Cell Biochem. Funct..

[18] Gimble J.M., Katz A.J., Bunnell B.A. (2007). Adipose-derived stem cells for regenerative medicine. Circ. Res..

[19] Schaffler A., Buchler C. (2007). Concise review: Adipose tissue-derived stromal cells--basic and clinical implications for novel cell-based therapies. Stem. Cells.

[20] Nakagami H., Maeda K., Morishita R., Iguchi S., Nishikawa T., Takami Y., Kikuchi Y., Saito Y., Tamai K., Ogihara T. (2005). Novel autologous cell therapy in ischemic limb disease through growth factor secretion by cultured adipose tissue-derived stromal cells. Arterioscler. Thromb. Vasc. Biol..

[21] Cherubino M., Rubin J.P., Miljkovic N., Kelmendi-Doko A., Marra K.G. (2011). Adipose-derived stem cells for wound healing applications. Ann. Plast. Surg..

[22] Onate B., Vilahur G., Ferrer-Lorente R., Ybarra J., Díez-Caballero A., Ballesta-López C., Moscatiello F., Herrero J., Badimon L. (2012). The subcutaneous adipose tissue reservoir of functionally active stem cells is reduced in obese patients. FASEB J..

[23] Perez L.M., Bernal A., San Martín N., Gálvez B.G. (2013). Obese-derived ASCs show impaired migration and angiogenesis properties. Arch. Physiol. Biochem..

[24] Camussi G., Deregibus M.C., Bruno S., Cantaluppi V., Biancone L. (2010). Exosomes/microvesicles as a mechanism of cell-to-cell communication. Kidney Int..

[25] Kang T., Jones T.M., Naddell C., Bacanamwo M., Calvert J.W., Thompson W.E., Bond V.C., Chen Y.E., Liu D. (2016). Adipose-Derived Stem Cells Induce Angiogenesis via Microvesicle Transport of miRNA-31. Stem Cells Transl. Med..

[26] Lambert C., Arderiu G., Bejar M.T., Crespo J., Baldellou M., Juan-Babot O., Badimon L. (2019). Stem cells from human cardiac adipose tissue depots show different gene expression and functional capacities. Stem Cell Res. Ther..

[27] Ferrer-Lorente R., Bejar M.T., Badimon L. (2014). Notch signaling pathway activation in normal and hyperglycemic rats differs in the stem cells of visceral and subcutaneous adipose tissue. Stem Cells Dev..

[28] Ferrer-Lorente R., Bejar M.T., Tous M., Vilahur G., Badimon L. (2014). Systems biology approach to identify alterations in the stem cell reservoir of subcutaneous adipose tissue in a rat model of diabetes: Effects on differentiation potential and function. Diabetologia.

[29] Bejar M.T., Ferrer-Lorente R., Peña E., Badimon L. (2016). Inhibition of Notch rescues the angiogenic potential impaired by cardiovascular risk factors in epicardial adipose stem cells. FASEB J..

[30] Onate B., Vilahur G., Camino-López S., Díez-Caballero A., Ballesta-López C., Ybarra J., Moscatiello F., Herrero J., Badimon L. (2013). Stem cells isolated from adipose tissue of obese patients show changes in their transcriptomic profile that indicate loss in stemcellness and increased commitment to an adipocyte-like phenotype. BMC Genom..

[31] Arderiu G., Peña E., Aledo R., Juan-Babot O., Crespo J., Vilahur G., Oñate B., Moscatiello F., Badimon L. (2019). MicroRNA-145 Regulates the Differentiation of Adipose Stem Cells Toward Microvascular Endothelial Cells and Promotes Angiogenesis. Circ. Res..

[32] Fish J.E., Santoro M.M., Morton S.U., Yu S., Yeh R.F., Wythe J.D., Ivey K.N., Bruneau B.G., Stainier D.Y., Srivastava D. (2008). miR-126 regulates angiogenic signaling and vascular integrity. Dev. Cell.

[33] Bian X., Ma K., Zhang C., Fu X. (2019). Therapeutic angiogenesis using stem cell-derived extracellular vesicles: An emerging approach for treatment of ischemic diseases. Stem Cell Res. Ther..

[34] Olshansky S.J., Passaro D.J., Hershow R.C., Layden J., Carnes B.A., Brody J., Hayflick L., Butler R.N., Allison D.B., Ludwig D.S. (2005). A potential decline in life expectancy in the United States in the 21st century. N. Engl. J. Med..

[35] Badimon L., Onate B., Vilahur G. (2015). Adipose-derived Mesenchymal Stem Cells and Their Reparative Potential in Ischemic Heart Disease. Rev. Esp. Cardiol. Engl. Ed..

[36] Becerra S.P., Notario V. (2013). The effects of PEDF on cancer biology: Mechanisms of action and therapeutic potential. Nat. Rev. Cancer.

[37] Cai J., Chen Z., Ruan Q., Han S., Liu L., Qi X., Boye S.L., Hauswirth W.W., Grant M.B., Boulton M.E. (2011). gamma-Secretase and presenilin mediate cleavage and phosphorylation of vascular endothelial growth factor receptor-1. J. Biol. Chem..

[38] He X., Cheng R., Benyajati S., Ma J.X. (2015). PEDF and its roles in physiological and pathological conditions: Implication in diabetic and hypoxia-induced angiogenic diseases. Clin. Sci..

[39] Hosseinzadeh-Attar M.J., Mahdavi-Mazdeh M., Yaseri M., Zahed N.S., Alipoor E. (2017). Comparative Assessment of Serum Adipokines Zinc-alpha2-glycoprotein and Adipose Triglyceride Lipase, and Cardiovascular Risk Factors Between Normal Weight and Obese Patients with Hemodialysis. Arch. Med. Res..

[40] Mairal A., Langin D., Arner P., Hoffstedt J. (2006). Human adipose triglyceride lipase (PNPLA2) is not regulated by obesity and exhibits low in vitro triglyceride hydrolase activity. Diabetologia.

[41] Steinberg G.R., Kemp B.E., Watt M.J. (2007). Adipocyte triglyceride lipase expression in human obesity. Am. J. Physiol. Endocrinol. Metab..

[42] Schramme A., Mussbacher M., Wölkart G., Stessel H., Pail K., Winkler S., Schweiger M., Haemmerle G., Al Zoughbi W., Höfler G. (2014). Endothelial dysfunction in adipose triglyceride lipase deficiency. Biochimica. Biophysica. Acta.

[43] Lee H.J. (2018). Exercise training regulates angiogenic gene expression in white adipose tissue. J. Exerc. Rehabil..

[44] Mazaki-Tovi S., Tarca A.L., Vaisbuch E., Kusanovic J.P., Than N.G., Chaiworapongsa T., Dong Z., Hassan S.S., Romero R. (2016). Characterization of visceral and subcutaneous adipose tissue transcriptome in pregnant women with and without spontaneous labor at term: Implication of alternative splicing in the metabolic adaptations of adipose tissue to parturition. J. Perinat. Med..

[45] Peinado J.R., Pardo M., de la Rosa O., Malagón M.M. (2012). Proteomic characterization of adipose tissue constituents, a necessary step for understanding adipose tissue complexity. Proteomics.

[46] Perez-Perez R., Ortega-Delgado F.J., García-Santos E., López J.A., Camafeita E., Ricart W., Fernández-Real J.M., Peral B. (2009). Differential proteomics of omental and subcutaneous adipose tissue reflects their unalike biochemical and metabolic properties. J. Proteome Res..

[47] Lemoine A.Y., Ledoux S., Larger E. (2013). Adipose tissue angiogenesis in obesity. Thromb. Haemost..

[48] Huang H., Bhat A., Woodnutt G., Lappe R. (2010). Targeting the ANGPT-TIE2 pathway in malignancy. Nat. Rev. Cancer.

[49] Linares P.M., Chaparro M., Gisbert J.P. (2014). Angiopoietins in inflammation and their implication in the development of inflammatory bowel disease. A review. J. Crohns Colitis.

[50] Abramovich D., Irusta G., Bas D., Cataldi N.I., Parborell F., Tesone M. (2012). Angiopoietins/TIE2 system and VEGF are involved in ovarian function in a DHEA rat model of polycystic ovary syndrome. Endocrinology.

[51] Penton A.L., Leonard L.D., Spinner N.B. (2012). Notch signaling in human development and disease. Semin. Cell Dev. Biol..

[52] Henshall T.L., Keller A., He L., Johansson B.R., Wallgard E., Raschperger E., Mäe M.A., Jin S., Betsholtz C., Lendahl U. (2015). Notch3 is necessary for blood vessel integrity in the central nervous system. Arterioscler. Thromb. Vasc. Biol..

[53] Niessen K., Karsan A. (2007). Notch signaling in the developing cardiovascular system. Am. J. Physiol. Cell Physiol..

[54] Pitulescu M.E., Schmidt I., Giaimo B.D., Antoine T., Berkenfeld F., Ferrante F., Park H., Ehling M., Biljes D., Rocha S.F. (2017). Dll4 and Notch signalling couples sprouting angiogenesis and artery formation. Nat. Cell Biol..

[55] Siekmann A.F., Lawson N.D. (2007). Notch signalling and the regulation of angiogenesis. Cell Adhes. Migr..

[56] Park S., Sorenson C.M., Sheibani N. (2015). PECAM-1 isoforms, eNOS and endoglin axis in regulation of angiogenesis. Clin. Sci..

[57] Sun L., Huang T., Xu W., Sun J., Lv Y., Wang Y. (2017). Advanced glycation end products promote VEGF expression and thus choroidal neovascularization via Cyr61-PI3K/AKT signaling pathway. Sci. Rep..

[58] Li B.K., Guo K., Li C.Y., Li H.L., Zhao P.P., Chen K., Liu C.X. (2015). Influence of suppression of CapG gene expression by siRNA on the growth and metastasis of human prostate cancer cells. Genet. Mol. Res..

[59] Silacci P., Mazzolai L., Gauci C., Stergiopulos N., Yin H.L., Hayoz D. (2004). Gelsolin superfamily proteins: Key regulators of cellular functions. Cell Mol. Life Sci..

[60] Turcot V., Bouchard L., Faucher G., Tchernof A., Deshaies Y., Pérusse L., Marceau P., Hould F.S., Lebel S., Vohl M.C. (2012). A polymorphism of the interferon-gamma-inducible protein 30 gene is associated with hyperglycemia in severely obese individuals. Hum. Genet..

[61] Benbow J.H., Thompson K.J., Cope H.L., Brandon-Warner E., Culberson C.R., Bossi K.L., Li T., Russo M.W., Gersin K.S., McKillop I.H. (2016). Diet-Induced Obesity Enhances Progression of Hepatocellular Carcinoma through Tenascin-C/Toll-Like Receptor 4 Signaling. Am. J. Pathol..

[62] Imanaka-Yoshida K., Yoshida T., Miyagawa-Tomita S. (2014). Tenascin-C in development and disease of blood vessels. Anat. Rec..

[63] Fan L., Wu Q., Xing X., Wei Y., Shao Z. (2012). MicroRNA-145 targets vascular endothelial growth factor and inhibits invasion and metastasis of osteosarcoma cells. Acta Biochim. Biophys. Sin..

[64] Sessa R., Seano G., di Blasio L., Gagliardi P.A., Isella C., Medico E., Cotelli F., Bussolino F., Primo (2012). L. The miR-126 regulates angiopoietin-1 signaling and vessel maturation by targeting p85beta. Biochim. Biophys. Acta.

[65] Ren S., Chen J., Duscher D., Liu Y., Guo G., Kang Y., Xiong H., Zhan P., Wang Y., Wang C. (2019). Microvesicles from human adipose stem cells promote wound healing by optimizing cellular functions via AKT and ERK signaling pathways. Stem Cell Res. Ther..

