# Supplementary material for: Cardiovascular Risk Factors and Differential Transcriptomic Profile of the Subcutaneous and Visceral Adipose Tissue and Their Resident Stem Cells
Source: Cells. 2020 Oct 3;9(10):2235. doi: 10.3390/cells9102235 (PMC7600037; doi:10.3390/cells9102235)
Supplement: Supplementary file 1 [file cells-09-02235-s001.pdf]

# Supplemental material

# Cardiovascular risk factors and differential transcriptomic profile of the subcutaneous and visceral adipose tissue and their resident stem cells

**Gemma Arderiu[1,3,§,*], Carmen Lambert[1,*], Carlos Ballesta[2], Fabrizio Moscatielo[2], G Vilahur[1,3], Lina Badimon[1,3,4,§]**

[1]Cardiovascular-Program ICCC, IR-Hospital Santa Creu I Sant Pau, IIB Sant Pau, Barcelona,

[2]Centro Médico Teknon, Barcelona, Spain;

[3]Ciber CV, Madrid, Spain;

[4]Cardiovascular Research Chair UAB; Barcelona, Spain.

§ Correspondence:

Dr. Gemma Arderiu, Cardiovascular Program IR-Hospital Santa Creu I Sant Pau, IIB Sant Pau, C/ Sant Antoni Mª Claret 167, 08025 Barcelona, Spain; Phone: (34) 935565880. Fax: (34) 935565559. garderiu@santpau.cat

Prof. Lina Badimon, Cardiovascular Program IR-Hospital Santa Creu I Sant Pau, IIB Sant Pau, C/ Sant Antoni Mª Claret 167, 08025 Barcelona, Spain; Phone: (34) 935565880. Fax: (34) 935565559. lbadimon@santpau.cat

**Supplemental Table S1:** TaqMan gene expression assay probes used in the custom array.

| Gene symbol | Gene name | TaqMan Assay reference | Localization |
|---|---|---|---|
| AGER | Advanced glycosilation end product-specific receptor | Hs00153957_m1 | Plasma membrane |
| ANGPT1 | Angiopoietin 1 | Hs00181613_m1 | Extracellular |
| ANGPT2 | Angiopoietin 2 | Hs00169867_m1 | Extracellular |
| CAPG | Macrophage-capping protein | Hs00156249_m1 | Cytoplasm, Nucleus, Secreted |
| CD34 | Hematopoietic progenitor dell antigen | Hs00156373_m1 | Plasma membrane |
| CD68 | Macrosialin | Hs00154355_m1 | Plasma membrane |
| COL18A1 | Collagen alpha-1(XVIII) chain | Hs00181017_m1 | Extracellular |
| DLK1 | Protein delta homolog 1 | Hs00171584_m1 | Plasma membrane |
| DLL4 | Delta like protein-4 | Hs00184092_m1 | Plasma membrane |
| ENG | Endoglin | Hs00923996_m1 | Plasma membrane |
| IFI30 | Gamma-intertefon-inducible lysosomal thiol reductase | Hs00173838_m1 | Lysosome, extracellulas region |
| NOTCH3 | Neurogenic locus notch homolog protein3 | Hs01128541_m1 | Plasma membrane |
| PECAM1 | Platelet endothelial cell adhesion molecule | Hs00169777_m1 | Plasma membrane |
| PGF | Placenta growth factor | Hs00182176_m1 | Extracellular |
| PLIN2 | Perlipin2 (PLIN2) | Hs00605340_m1 | Membrane, lipid droplets |
| PNPLA2 | Patatin-like phospholipase domain-containing protein 2 | Hs00386101_m1 | Membrane, lipid droplets |
| PTX3 | Pentraxin-related protein PTX3 | Hs00173615_m1 | Extracellular |
| SERPINF1 | Pigment epithelium-derived factor | Hs01106937_m1 | Extracellular |
| SPP1 | Osteopontin | Hs00959010_m1 | Extracellular |
| TEK | Angiopoietin-1 receptor | Hs00945155_m1 | Plasma membrane, extracellular |
| TNC | Tenascin | Hs01115665_m1 | Extracellular |
| VEGFA | Vascular endothelial growth factor A | Hs00900055_m1 | Extracellular |

**Supplemental Table S2:** Antibodies used to perform the experiments.

| | | |
|---|---|---|
| **CD105 PE anti-Human** | **BD** | **560839** |
| **CD44 PE anti-Human** | **BD PHARMINGEN** | **555479** |
| CD29 PE Mouse Anti-Human | BD | 555443 |
| CD90 FITC anti-Human | BD PHARMINGEN | 555595 |
| CD73 FITC anti-Human | BD PHARMINGEN | 561254 |
| CD45 PE-Cy5 anti-Human | BD PHARMINGEN | 555484 |
| CD14 FITC anti-Human | IMMUNOSTEP | 14F-100T |
| CFBlue Annexin V | IMMUNOSTEP | ANXVCFB |
| FITC-Conjugated Monoclonal Antibody Against Human Tissue Factor | CIENTISOL | 4508CJ |
| TF anti-goat | CIENTISOL | 4501 |
| anti beta-actin anti-mouse | ABCAM | ab8226 |
| VEGF Receptor2 (55B11) | CELL SIGNALING | 2479 |
| VEGFA | ABCAM | ab214424 |

**Supplemental Table S3:** Gene expression profile of the subcutaneous and visceral adipose tissue of patients and controls.

| | Obese | | Non-obese |
|---|---|---|---|
| | SAT ($2^{-\Delta CT}$)*1000 | VAT ($2^{-\Delta CT}$)*1000 | SAT ($2^{-\Delta CT}$)*1000 |
| **AGER** | 7.05 ± 0.64 | 8.30 ± 0.48 | 4.03 ± 0.53 |
| **ANGPT1** | 89.58 ± 9.17 | 52.7 ± 5.16 | 68.47 ± 5.30 |
| **ANGPT2** | 15.35 ± 1.93 | 8.89 ± 0.92 | 11.01 ± 1.01 |
| **CAPG** | 297.94 ± 24.63 | 254.41 ± 13.81 | 224.31 ± 16.15 |
| **CD34** | 349.26 ± 28.80 | 320.7 ± 22.89 | 250.38 ± 27.62 |
| **CD68** | 635.13 ± 41.73 | 515.71 ± 27.11 | 298.78 ± 49.20 |
| **COL18A1** | 244.05 ± 17.61 | 220.96 ± 11.44 | 262.04 ± 17.95 |
| **DLL4** | 36.47 ± 2.98 | 28.043 ±2.52 | 59.22 ± 6.77 |
| **ENG** | 354.15 ± 31.40 | 295.37 ± 13.72 | 382.82 ± 30.05 |
| **IFI30** | 288.80 ± 28.28 | 246.45 ± 22.96 | 113.32 ± 24.85 |
| **NOTCH3** | 337.38 ± 36.22 | 84.68 ± 6.18 | 198.90 ± 18.99 |
| **PECAM1** | 497.34 ± 49.34 | 327.96 ±21.81 | 562.18 ± 71.80 |
| **PGF** | 6.82 ± 0.76 | 8.26 ± 0.48 | 6.67 ± 1.10 |
| **PLIN2** | 297.58 ± 27.13 | 257.68 ± 14.46 | 442.55 ± 29.19 |
| **PNPLA2** | 1084 ± 62.10 | 831.19 ± 70.16 | 1747.56 ± 11017 |
| **PTX3** | 6.02 ± 2.37 | 5.44 ± 0.58 | 5.99 ± 1.39 |
| **SERPINF1** | 2562.60 ± 170.68 | 1705.26 ± 126.53 | 2459.77 ± 184.53 |
| **SPP1** | 238.56 ± 42.88 | 82.34 ± 12.96 | 50.08 ± 16.78 |
| **TEK** | 65.82 ± 4.17 | 53.02 ± 3.24 | 79.95 ± 8.54 |
| **TNC** | 38.79 ± 7.41 | 12.82 ± 1.11 | 34.56 ± 11.77 |
| **VEGFA** | 198.17 ± 20.14 | 231.32 ± 13.68 | 426.72 ± 54.49 |

**Supplemental Table S4.** Gene *expression* of SAT and VAT from non diabetic (non-DM) and diabetic (DM) obese patients. Significance between non-DM *versus* DM.

| | SUBCUTANEOUS | | | VISCERAL | | |
|---|---|---|---|---|---|---|
| **Gene** | **non-DM ($2^{-\Delta CT}$)*1000** | **DM ($2^{-\Delta CT}$)*1000** | **non-DM *vs* DM P-value** | **non-DM ($2^{-\Delta CT}$)*1000** | **DM ($2^{-\Delta CT}$)*1000** | **non-DM *vs* DM P value** |
| **AGER** | 7.12 ± 0.74 | 6.80 ± 1.37 | 0.906 | 8.31 ± 0.51 | 8.26 ± 1.36 | 0.969 |
| **ANGPT1** | 97.66 ± 10.79 | 62.12 ± 10.71 | 0.072 | 60.38 ± 6.46 | 46.66 ± 3.18 | 0.256 |
| **ANGPT2** | 16.52 ± 2.31 | 11.36 ± 2.91 | 0.127 | 8.89 ± 1.18 | 8.88 ± 0.70 | 0.411 |
| **CAPG** | 292.25 ± 26.21 | 317.3067.46 | 0.724 | 250.95 ± 14.53 | 266.19 ± 38.60 | 0.368 |
| **CD34** | 365.63 ± 32.28 | 293.59 ± 63.12 | 0.292 | 303.5 ± 21.35 | 379.19 ± 69.25 | 0.368 |
| **CD68** | 625.10 ± 44.51 | 669.24 ± 113.62 | 0.505 | 504.91 ± 28.68 | 552.45 ± 73.19 | 0.724 |
| **COL18A1** | 238.25 ± 17.62 | 263.75 ± 53.09 | 0.457 | 219.32 ± 12.09 | 226.53 ± 32.03 | 0.969 |
| **DLL4** | 38.23 ± 3.44 | 30.49 ± 5.73 | 0.255 | 27.26 ± 3.05 | 30.70 ± 4.28 | 0.368 |
| **ENG** | 347.79 ± 36.59 | 375.8 ± 66.43 | 0.092 | 283.80 ± 15.45 | 334.73 ± 24.48 | 0.092 |
| **IFI30** | 273.79 ± 26.54 | 339.84 ± 89.71 | 0.505 | 243.49 ± 26.54 | 256.51 ± 50.60 | 0.784 |
| **NOTCH3** | 329.73 ± 44.53 | 363.38 ± 55.81 | 0.327 | 84.78 ± 7.92 | 84.34 ± 5.46 | 0.906 |
| **PECAM1** | 500.98 ± 57.45 | 484.94 ± 106.32 | 0.906 | 320.66 ± 26.01 | 352.78 ± 39.80 | 0.365 |
| **PGF** | 6.62 ± 0.72 | 7.51 ± 2.49 | 0.906 | 8.10 ± 0.54 | 8.80 ± 1.13 | 0.457 |
| **PLIN2** | 280.11 ± 25.91 | 356.97 ± 82.03 | 0.457 | 247.77 ± 13.05 | 291.36 ± 46.30 | 0.417 |
| **PNPLA2** | 1120.28 ± 54.25 | 964.90 ± 210.66 | 0.557 | 867.77 ± 86.72 | 706.82 ± 80.22 | 0.457 |
| **PTX3** | 6.47 ± 3.05 | 4.50 ± 1.78 | 0.611 | 5.32 ± 0.71 | 5.86 ± 0.88 | 0.411 |
| **SERPINF1** | 2715.24 ± 196.51 | 2043.64 ± 246.24 | 0.092 | 1782.30 ± 158.90 | 1443.35 ± 65.09 | 0.368 |
| **SPP1** | 195.23 ± 31.68 | 385.85 ± 147.71 | 0.290 | 74.20 ± 15.87 | 110.02 ± 14.47 | 0.055 |
| **TEK** | 64.84 ± 4.79 | 69.15 ± 14.05 | 0.611 | 51.69 ± 4.09 | 57.54 ± 2.86 | 0.365 |
| **TNC** | 38.66 ± 9.10 | 39.25 ± 11.94 | 0.784 | 11.84 ± 1.08 | 16.14 ± 3.01 | 0.256 |
| **VEGFA** | 205.44 ± 23.19 | 173.46 ± 43.11 | 0.667 | 222.89 ± 14.09 | 259.96 ± 36.80 | 0.290 |

**Supplemental Table S5.** Gene *expression* of SAT and VAT from non dyslipemic (non-DLP) and dyslipemic (DLP) obese patients. Significance between non-DLP *versus* DLP.

| | SUBCUTANEOUS | | | VISCERAL | | |
|---|---|---|---|---|---|---|
| **Gene** | **non-DLP ($2^{-\Delta CT}$)*1000** | **DLP ($2^{-\Delta CT}$)*1000** | **non-DLP *vs* DLP P-value** | **non-DLP ($2^{-\Delta CT}$)*1000** | **DLP ($2^{-\Delta CT}$)*1000** | **non-DLP *vs* DLP P value** |
| **AGER** | 6.38 ± 0.56 | 7.72 ± 1.14 | 0.375 | 8.58 ± 0.67 | 8.02 ± 0.71 | 0.718 |
| **ANGPT1** | 99.31 ± 10.92 | 79.85 ± 14.66 | 0.076 | 63.65 ± 9.4 | 50.87 ± 3.9 | 0.279 |
| **ANGPT2** | 12.22 ± 1.51 | 18.48 ± 3.39 | 0.178 | 9.71 ± 1.68 | 8.07 ± 0.75 | 0.622 |
| **CAPG** | 292.66 ± 33.87 | 303.23 ± 37.35 | 0.87 | 237.78 ± 19.61 | 271.04 ± 19.01 | 0.200 |
| **CD34** | 321.84 ± 30.3 | 376.67 ± 49.15 | 0.412 | 310.62 ± 37.44 | 330.78 ± 27.89 | 0.412 |
| **CD68** | 628.63 ± 54.92 | 641.63 ± 65.49 | 0.922 | 559.19 ± 45.88 | 472.23 ± 24.58 | 0.178 |
| **COL18A1** | 210.01 ± 12.87 | 278.09 ± 30.09 | **0.033** | 227 ± 17.77 | 214.93 ± 15.05 | 0.718 |
| **DLL4** | 31.37 ± 4.11 | 41.57 ± 3.9 | 0.061 | 26.63 ± 4.61 | 29.46 ± 2.24 | 0.158 |
| **ENG** | 309.31 ± 15.67 | 399 ± 59.1 | 0.123 | 284.18 ± 23.59 | 306.56 ± 14.45 | 0.450 |
| **IFI30** | 281.41±33.01 | 296.18 ± 47.51 | 0.768 | 271.26 ± 36.59 | 221.64 ± 27.42 | 0.309 |
| **NOTCH3** | 264.52 ± 23.1 | 410.23 ± 62.56 | 0.071 | 81.19 ± 9.96 | 88.18 ± 7.67 | 0.622 |
| **PECAM1** | 439.63 ± 27.2 | 555.04 ± 93.92 | 0.450 | 340.81 ± 38.92 | 315.11 ± 21.21 | 0.818 |
| **PGF** | 5.54 ± 0.61 | 8.1 ± 1.32 | 0.123 | 8.1 ± 0.69 | 8.42 ± 0.7 | 0.670 |
| **PLIN2** | 316.24 ± 39.11 | 278.91 ± 38.62 | 0.870 | 245.07 ± 20.76 | 270.29 ± 20.37 | 0.279 |
| **PNPLA2** | 1165.41 ± 81.6 | 1004.53 ± 90.79 | 0.341 | 843.06 ± 118.89 | 819.32 ± 80.7 | 0.718 |
| **PTX3** | 2.66 ± 0.67 | 9.38 ± 4.58 | 0.622 | 5.47 ± 0.96 | 5.42 ± 0.7 | 0.870 |
| **SERPINF1** | 2573.44 ± 235.73 | 2551.76 ± 258.39 | 0.974 | 1747.51 ± 231.96 | 1663.02 ± 114.38 | 0.533 |
| **SPP1** | 190.05 ± 37.93 | 287.06 ± 76.25 | 0.622 | 74.67 ± 19.25 | 90.01 ± 17.98 | 0.341 |
| **TEK** | 62.71 ± 5.92 | 68.93 ± 7.51 | 0.450 | 53.57 ± 5.72 | 52.47 ± 3.34 | 0.870 |
| **TNC** | 30.96 ± 6.72 | 46.62 ± 13.16 | 0.412 | 11.83 ± 1.11 | 13.8 ± 1.94 | 0.768 |
| **VEGFA** | 194.1 ± 21.6 | 202.24 ± 35.12 | 0.768 | 230.42 ± 24.19 | 232.22 ± 14.15 | 0.670 |

**Supplemental Table S6.** Gene *expression* of SAT and VAT from non hypertens (non-HP) and hypertens (HP) obese patients. Significance between non-HP *versus* HP.

| | SUBCUTANEOUS | | | VISCERAL | | |
|---|---|---|---|---|---|---|
| **Gene** | **non-DLP ($2^{-\Delta CT}$)*1000** | **DLP ($2^{-\Delta CT}$)*1000** | **non-DLP *vs* DLP P-value** | **non-DLP ($2^{-\Delta CT}$)*1000** | **DLP ($2^{-\Delta CT}$)*1000** | **non-DLP *vs* DLP P value** |
| **AGER** | 6.89±0.63 | 7.4±1.57 | 0.860 | 7.99±0.6 | 8.61±0.77 | 0.698 |
| **ANGPT1** | 101.59±9.99 | 77.58±14.98 | **0.007** | 62.55±9.53 | 51.96±3.91 | 0.972 |
| **ANGPT2** | 13.22±1.71 | 17.48±3.44 | 0.459 | 9.02±1.55 | 8.76±1.07 | 0.459 |
| **CAPG** | 303.05±33.52 | 292.84±37.67 | 0.597 | 253.76±19.66 | 255.06±20.36 | 0.459 |
| **CD34** | 330.51±28.51 | 368±51 | 0.805 | 321.63±35.65 | 319.77±30.48 | 0.062 |
| **CD68** | 626.47±55.72 | 643.79±64.76 | 0.647 | 573.5±41.87 | 457.92±25.82 | 0.217 |
| **COL18A1** | 220.47±17.25 | 267.62±29.9 | 0.459 | 229.86±18.38 | 212.06±14 | 0.549 |
| **DLL4** | 33.57±4.23 | 39.37±4.22 | 0.805 | 22.81±2.82 | 33.28±3.64 | 0.045 |
| **ENG** | 312.04±16.8 | 396.27±59.19 | 0.148 | 268.59±18.91 | 322.16±17.01 | 0.053 |
| **IFI30** | 279.69±32.93 | 297.91±47.51 | 0.418 | 287.02±34.88 | 205.87±25.85 | 0.148 |
| **NOTCH3** | 254.68±22.97 | 420.08±60.11 | 0.113 | 73.13±8.24 | 96.24±8.12 | 0.459 |
| **PECAM1** | 465.39±34.97 | 529.29±93.8 | 0.751 | 322.51±35.39 | 333.4±27.18 | 0.307 |
| **PGF** | 6.1±0.78 | 7.54±1.31 | 0.098 | 8.4±0.81 | 8.12±0.54 | 0.972 |
| **PLIN2** | 333.73±39.39 | 261.42±35.75 | 0.698 | 256.82±22.75 | 258.53±18.97 | 0.192 |
| **PNPLA2** | 1209.45±77.95 | 960.49±83.78 | 0.698 | 862.61±123.57 | 799.77±72.16 | 0.307 |
| **PTX3** | 6±3.45 | 6.05±3.43 | 0.148 | 5.33±0.98 | 5.56±0.67 | 0.698 |
| **SERPINF1** | 2671.73±211.38 | 2453.48±274.4 | 0.275 | 1781.31±226.49 | 1629.22±121.6 | 0.698 |
| **SPP1** | 205.37±35.63 | 271.74±78.96 | 0.459 | 60.24±12.02 | 104.44±21.53 | 0.140 |
| **TEK** | 65.64±5.8 | 66±7.72 | 0.751 | 51.64±5.86 | 54.41±3.05 | 0.378 |
| **TNC** | 29.88±6.21 | 47.7±13.28 | **0.032** | 11.25±1.04 | 14.39±1.9 | 0.418 |
| **VEGFA** | 206.1±20.72 | 190.24±35.52 | 0.805 | 238.96±23.92 | 223.68±14.22 | 0.916 |

**Supplemental Figure S1**

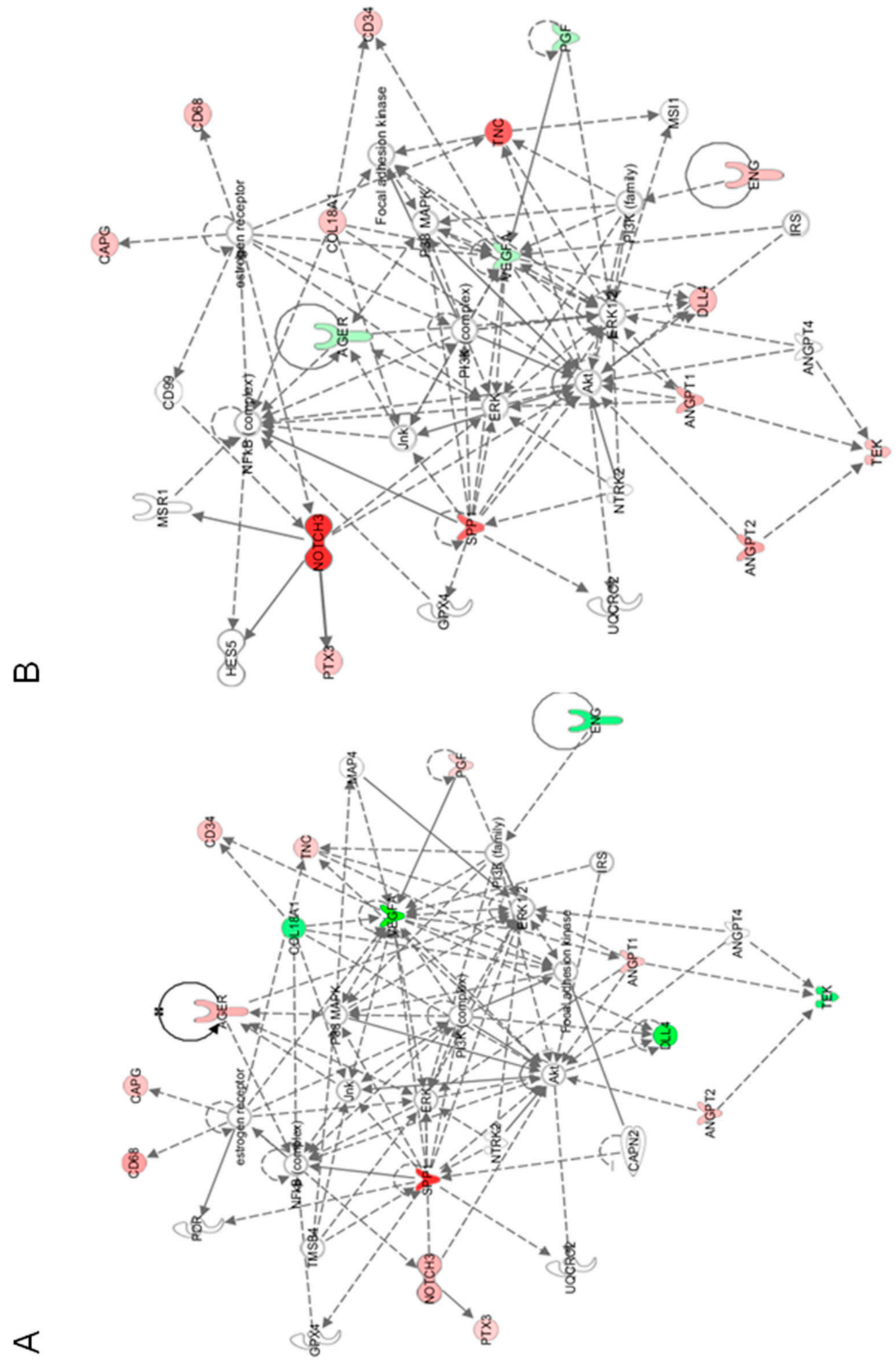


(A) IPA network analysis of target genes in subcutaneous adipose tissue from obese and non obese patients. (B) IPA network analysis of target genes in subcutaneous or visceral adipose tissue.

**Supplemental Figure S2**

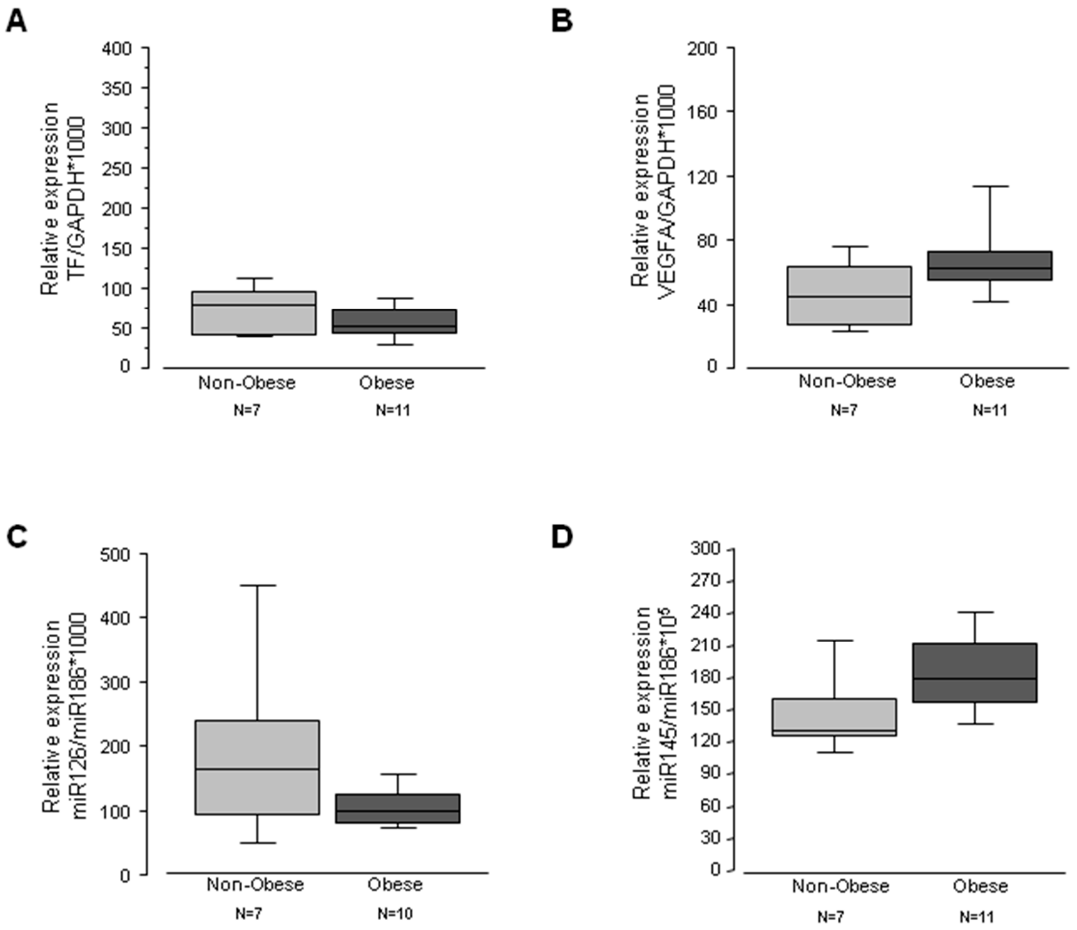


Box-plot diagram showing ASC genomic profile. A) Differential protein expression of TF in sASC from obese vs non-obese patients. B) Differential protein expression of VEGFA in sASC from obese vs non-obese patients. C) Differential protein expression of miR126 in sASC from obese vs non-obese patients. D) Differential protein expression of miR145 in sASC from obese vs non-obese patients.
